# Prioritizing Candidate YB‑1 Cold Shock Domain Ligands via 5D-ElectroShape and Boltz Generative Cofolding

**DOI:** 10.1021/acsomega.6c02840

**Published:** 2026-08-14

**Authors:** Lalehan Oktay, Cem Uğuz, Serdar Durdağı

**Affiliations:** † Computational Biology and Molecular Simulations Laboratory, Department of Biophysics, School of Medicine, 52946 Bahçeşehir University, Istanbul 34734, Türkiye; ‡ Lab for Innovative Drugs (Lab4IND), Computational Drug Design Center (HİTMER), Bahçeşehir University, Istanbul 34734, Türkiye; § Neuroscience Program, Graduate School, Bahçeşehir University, Istanbul 34734, Türkiye; ∥ Quantitative System Biology Lab, Faculty of Medicine, Biruni University, Istanbul 34015, Türkiye; ⊥ Molecular Therapy Lab, Department of Pharmaceutical Chemistry, School of Pharmacy, Bahçeşehir University, Istanbul 34353, Türkiye

## Abstract

Y-box binding protein 1 (YB-1) acts as a key oncoprotein, with its highly conserved cold shock domain (CSD) driving core nucleic acid-binding functions in cancer progression. Despite its therapeutic relevance, the CSD is considered “undruggable” due to the lack of a well-defined hydrophobic pocket. To overcome the limitations of rigid docking in this transient pocket, here, we present a computational ligand-prioritization workflow designed to generate experimentally testable hypotheses for this challenging target. We applied the 5D-ElectroShape formalism combined with our in-house MolPrism clustering toolkit to compress a library of ∼11,000 RNA-protein interaction–focused small molecules into 70 representative diversity centroids. These candidates were subjected to *ab initio* complex prediction using the deep learning-based protein folding algorithm Boltz-1, which successfully modeled the induced-fit requirements of the CSD cryptic pocket. A structure-driven selection strategy, utilizing AlphaFold-derived confidence metrics (*ipTM* ≥ 0.88), prioritized 18 high-confidence candidate complexes for further analysis. Subsequent molecular dynamics (MD) simulations and MM/GBSA binding free-energy calculations were used to prioritize compounds that maintained stable pocket engagement under dynamic conditions. Among the prioritized compounds, F3382–0454 showed stable cryptic-pocket occupancy during MD simulations and ligand-efficiency values comparable to known YB-1 inhibitor SU056, supporting its selection as a top-ranked candidate scaffold for future testing. Taken together, these results support the use of chemical-space compression followed by generative cofolding and MD-based refinement as a practical workflow for generating hypotheses about cryptic YB-1 ligands, while experimental validation and prospective benchmarking remain necessary.

## Graphical abstract

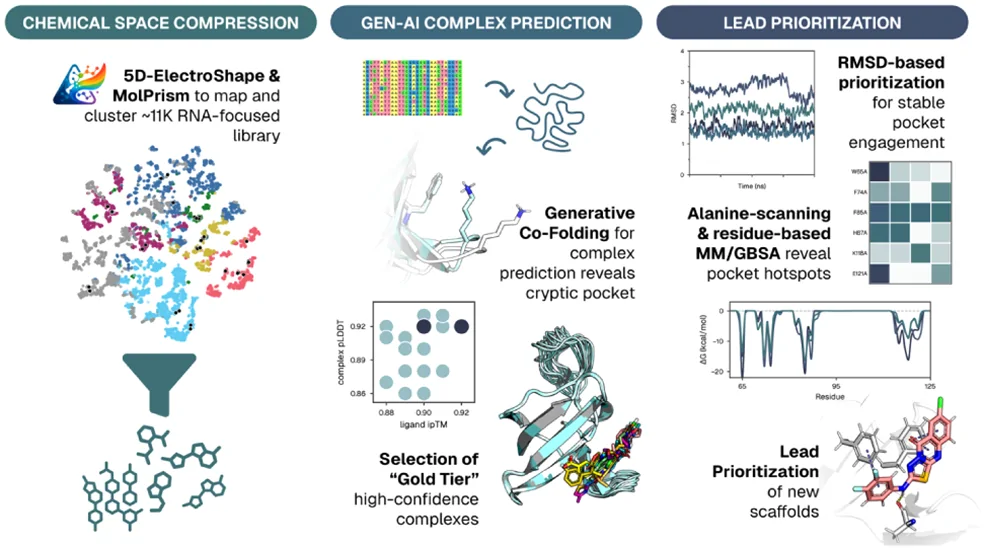

## 1 Introduction

The Y-box binding protein 1 (YB-1) is a DNA/RNA-binding protein that acts as a master regulator of transcription and translation.^1^ Its overexpression is associated with poor patient outcome, resulting in higher relapse rates and reduced survival in various cancers, driving tumor cell proliferation, invasion, migration, and metastasis.^2–7^ The protein contains three distinct domains: the Alanine/Proline (A/P)-rich N-terminal disordered domain, the disordered C-terminal domain, and a highly conserved and well-folded nucleic acid-binding Cold Shock Domain (CSD).^8^ The CSD forms a β-barrel OB-fold consisting of five antiparallel β-sheets.^9^ Despite its therapeutic relevance, targeting the CSD remains challenging due to the lack of a well-defined hydrophobic binding pocket. In the CSD, the two common binding regions, RNP1 (ribonucleoprotein domain 1) (W65, F74) and RNP2 (ribonucleoprotein domain 2) (F85, H87), both engage in flat aromatic binding and are solvent-exposed, making them challenging to target with small molecules.^10^

One of the earliest approaches to target YB-1 with small molecules proposed that the flavonoid fisetin, a dual inhibitor of the PI3K/Akt and mTOR signaling pathways, would also bind to the YB-1 CSD. This hypothesis was supported by molecular docking studies of fisetin to the proposed binding pocket located within the β-barrel of the YB-1 CSD. The binding was further validated through Surface Plasmon Resonance (SPR) experiments, which revealed a binding affinity of 35 μM for fisetin to YB-1.^11^ In another study, Senarisoy et al.^12^ identified inhibitors of the protein–protein interaction between human NTH1 (endonuclease III) and YB-1. FDA-approved oxytetracycline and meclocycline were found to sensitize drug-resistant MCF-7 cells to paclitaxel. These compounds, among others, induced noticeable thermal shift (ΔT_m_) of YB-1ΔC, a YB-1 construct lacking the C-terminal domain, indicating a direct interaction with YB-1 itself. Meclocycline induced two peaks (ΔT_m_ ≈ 2.3 °C and ΔT_m_ ≈ 5.55 °C), and oxytetracycline also induced two peaks at (ΔT_m_ ≈ 0.3 °C and ΔT_m_ ≈ 5.43 °C), suggesting the presence of potential binding sites on YB-1.^12^

In a recent study, Tailor et al.^13^ identified SU056, an azopodophyllotoxin derivative, as a potent YB-1 inhibitor in ovarian cancer cells through rational drug design and bioisostere replacement lead optimization. Target identification of this compound, performed via Cellular Thermal Shift Assays (CETSA) coupled with liquid chromatography-tandem mass spectrometry (LC-MS/MS), revealed YB-1 as its primary interactor among the 804 profiled proteins, displaying the largest thermal shift (ΔT_m_ = 5.92 °C ± 0.86 °C). This direct interaction was further validated using pull-down assays and SPR.^13^ A significant structural breakthrough occurred when El Hage et al.^14^ demonstrated via solution NMR spectroscopy (^1^H–^15^N SOFAST-HMQC) and molecular dynamics (MD) simulations that the fisetin binding pocket proposed by Khan et al.^11^ was incorrect. Instead, they showed that fisetin specifically engaged residues in a newly identified hydrophobic pocket on the surface of the β-barrel. The accessibility of this pocket is governed by a dynamic mechanism mediated by the aromatic anchor F85 and the cationic ammonium side chain of K118. El Hage et al.^14^ subsequently identified Niraparib, an FDA-approved poly­(ADP-ribose) polymerase (PARP) inhibitor, through an exhaustive *in silico* pipeline, as a potent YB-1 inhibitor. They validated its direct binding to the protein by NMR spectroscopy and demonstrated its ability to disrupt YB-1/mRNA interactions in cells using a newly developed screening method, the Microtubule (MT) Bench assay.^14^

The discovery of this dynamic, cryptic pocket and the conventionally hard to target nature of YB-1 suggests that static representations of YB-1 are insufficient for structure-based drug design. To accurately capture the induced-fit changes of the cryptic pocket required for ligand binding, advanced modeling approaches capable of predicting protein flexibility are necessary. While deep learning (DL)-based protein folding tools like AlphaFold3 and Boltz-1 have revolutionized structural biology, applying them to high-throughput screening of large libraries (10,000+ compounds) remains computationally challenging. To gain the benefits of generative AI-based protein–ligand interaction prediction without the computational expense, we implemented a ligand-based dimensionality reduction strategy. We utilized an implementation of the 5D-ElectroShape method proposed by Armstrong et al.^15,16^ The implementation of the charge (q) and lipophilicity (l) dimensions in the 5D formalism is critical for targeting the CSD, as distinguishing between steric isosteres is necessary to identify new chemotypes capable of engaging the specific F85/K118 cryptic pocket. By combining this with our in-house MolPrism clustering algorithm, we compressed the chemical space of our curated library of compounds targeting RNA-protein interactions. This allowed the selection of a manageable, structurally diverse subset for high-accuracy complex prediction using Boltz-1, aiming to prioritize candidate chemotypes targeting the YB-1 CSD, which were then subsequently evaluated through all-atom MD simulations and binding free-energy calculations.

The objective of the present work is to evaluate whether a hierarchical workflow combining 5D-ElectroShape-based chemical-space compression, Boltz-1 cofolding, and MD refinement could prioritize additional chemotypes compatible with the previously described F85/K118-gated cryptic pocket. We therefore frame this study as a computational prioritization strategy intended to enrich plausible binders in a tractable subset of compounds.

## 2 Results and Discussion

### 2.1 Rationale for 5D-ElectroShape Implementation and Chemical Space Coverage with MolPrism

The 5D-ElectroShape method is essential for overcoming the limitations of traditional virtual screening for “undruggable” targets. In contrast to conventional shape-based approaches, the 5D-ElectroShape method extends the standard 3D Cartesian coordinate system by incorporating charge (q) and atomic lipophilicity (l) as the fourth and fifth dimensions, respectively.^15,16^ For the YB-1 CSD, this distinction is critical since the identified binding site is a dynamic, cryptic pocket influenced by the aromatic side chain of F85 and the cationic side chain of K118. Therefore, a ligand must not only geometrically fit but also possess the precise l and q distributions to engage these specific residues. The generated feature vector was used for clustering with our in-house Molecular Clustering and Analysis (MolPrism) toolkit.

A focused collection of 11,594 molecules was curated from RNA-associated small molecule libraries and then we employed an automated K-means clustering algorithm, resulting in 7 clusters and 10 representative structures per cluster. We hypothesize that the 70 representatives from the 7 clusters provide a broad and computationally manageable sampling of the library’s 5D-ElectroShape descriptor space. To validate the chemical space coverage of the selected clusters, a t-distributed Stochastic Neighbor Embedding (t-SNE) was performed on the 18-dimensional 5D-ElectroShape descriptor vectors. The projection reveals a well-defined descriptor space where the K-means algorithm effectively uncovered seven distinct chemotype islands. By selecting 10 representative centroids per cluster (Figure 1A), we sought to ensure that the subsequent generative folding phase sampled a wide range of stereoelectronic features present in the curated library. Furthermore, we also analyzed the AlogP (Ghose-Crippen) distribution across the clusters (Figure 1B) and confirmed that the MolPrism-derived centroids accurately sample the lipophilic variance of the library, thereby ensuring the physicochemical diversity. We note that the t-SNE and AlogP analyses are intended as qualitative evidence of descriptor-space diversity.

**Figure 1:**
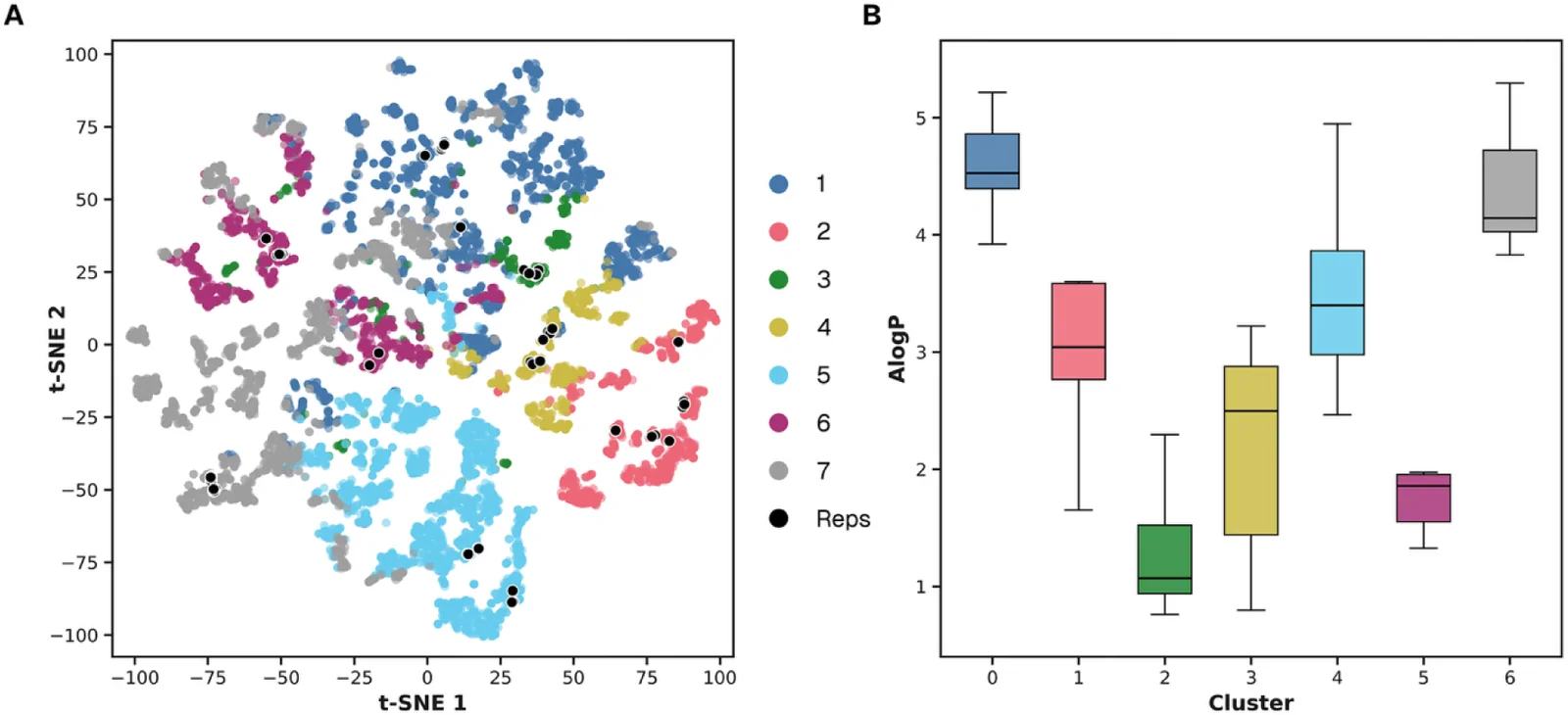
Rationale for 5D-ElectroShape and MolPrism A) t-SNE projection of the clustered chemical space B) AlogP box and whisker plot of clustered chemical space.

### 2.2 From Conventional Docking to Generative Cofolding

Before adopting Boltz-1 generative cofolding, we had attempted conventional docking using the grid-based Glide/SP algorithm^17^ of SU056 on the YB-1 CSD receptor prepared from PDB 6KUG^18^ with the cocrystallized 4-mer RNA excised, using four docking grids centered on the RNA-contact subsites identified by Yang et al.;^9^ F85/K118 (A2 subsite, the cryptic pocket itself), W65/F74/D83 (U3 subsite), W65/N67 (C4 subsite), and H87/E121 (C1 subsite). The top-ranked Glide/SP pose from each grid was then subjected to three independent 1 μs MD simulations replicas, yielding 12 μs of aggregate MD simulation time and 24,000 trajectory frames. To assess the structural stability of ligands at the binding pocket, we monitored the center-of-mass (CoM) distance between SU056 and the side-chain centers of mass of F74 and K118. The rationale behind choosing F74 as the aromatic probe comes from our observation that SU056 formed persistent π-stacking interactions with F74 throughout the productive replicas. Across all 12 μs of trajectories, only the W65/N67 (2/3 replicas) and W65/F74/D83 (1/3 replicas)-centered grids produced sustained occupancy of the F85/K118 cryptic pocket, and only via on-surface diffusion that required the K118/F85 cation-π gate to open during the simulations. Interestingly, the grid centered on the cryptic pocket itself produced the most rapid dissociation. Thus, the static docking score ranking was anticorrelated with this MD outcome, with the worst-scoring grid yielding the only sustained binding trajectories. This discrepancy likely reflects the strong dependence of the cryptic pocket geometry on receptor state. Although the pocket is open in the RNA-bound 6KUG structure, its side-chain rotamers are templated by RNA contacts. After RNA removal, these rotamers relax during MD, causing docking poses anchored to the RNA-stabilized geometry to drift from their initial configuration. Consistent with this interpretation, the alternative apo state, PDB 6LMS^10^ adopts a closed state in which the K118/F85 cation−π interaction is engaged. By contrast, Boltz-1 cofolding produces an initial structure in which the binding pocket is already adjusted to the ligand, and this conformation remains stable under the same MD conditions. The complete distance-trace data and docking score table are provided in Supplementary Figure S1 and Supplementary Table S1, respectively.

### 2.3 Correctly Targeting the F85/K118 Cryptic Pocket with Boltz

A major challenge in targeting YB-1 is the absence of a high-resolution ligand-bound crystal structure that defines a druggable binding mode. The cryptic binding site of interest, previously characterized by El Hage et al.,^14^ is regulated by a dynamic gating mechanism involving the aromatic residue F85 and the cationic residue K118. Such transient and conformationally restricted sites are often poorly sampled by conventional rigid-receptor docking protocols, which typically rely on a preformed binding cavity and limited receptor flexibility. In this context, our results indicate that the DL-based structure prediction framework Boltz-1 can capture a ligand-compatible conformational state of this dynamically gated pocket during protein–ligand complex prediction (Figure 2).

**Figure 2:**
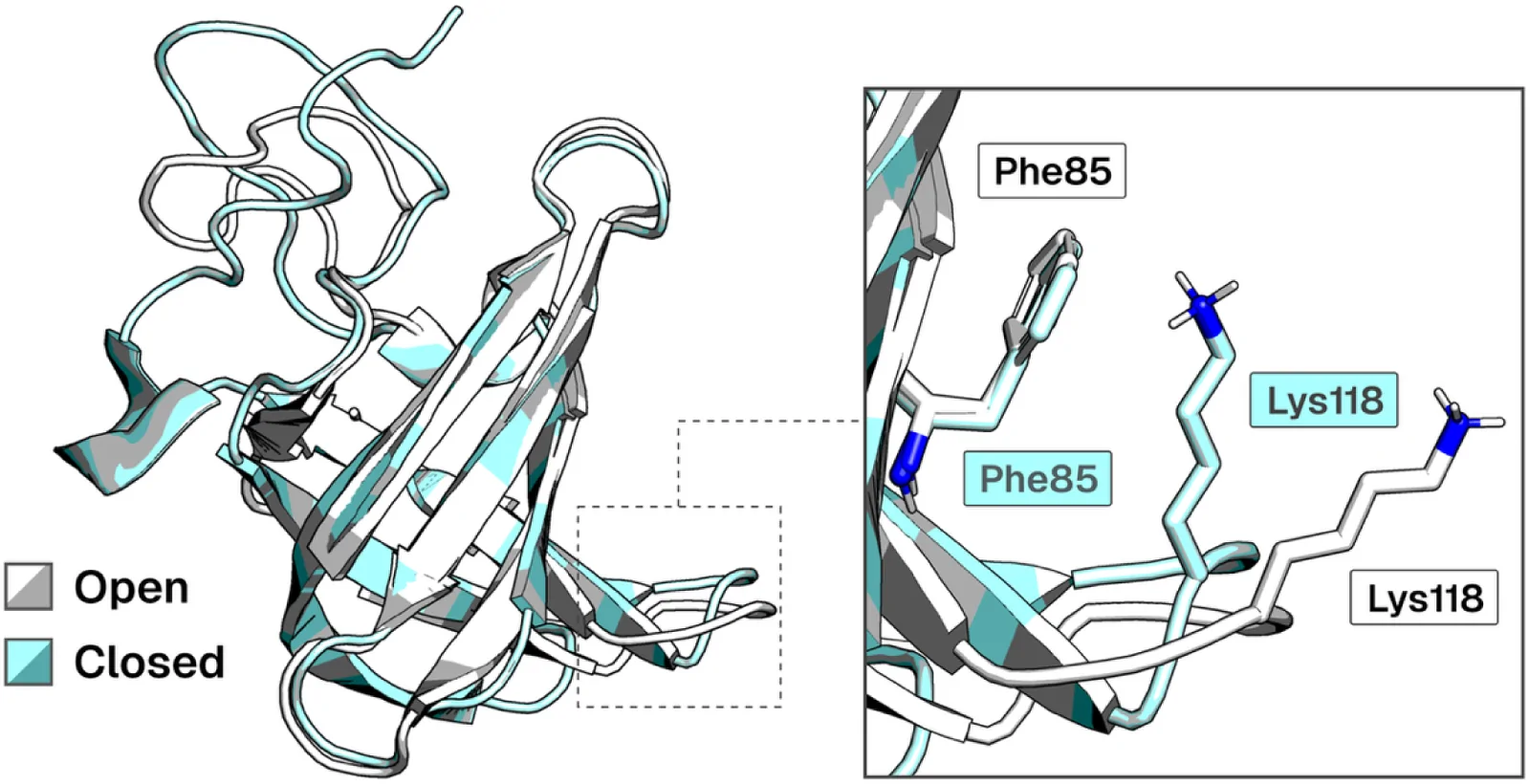
Open and closed conformation of YB-1 cryptic pocket, PDB 6LMS^10^ is given in cyan and Boltz-1 predicted model in white.

Taken together, these findings suggest that Boltz-1 can provide structurally plausible initial binding models for cryptic YB-1 pockets, which can subsequently be evaluated and refined through all-atom MD simulations to assess pocket stability, ligand retention, and induced-fit rearrangements.

### 2.4 Selection of High-Confidence Complexes

Boltz-1 successfully generated folded complexes for the selected ligands. All of these ligands were predicted to occupy the cryptic pocket of the YB-1 CSD. After generating these 71 protein–ligand complexes (70 focused compounds and 1 positive control compound SU056), we established a filtering protocol to identify the most promising candidates for further MD simulations. We opted toward a structure-driven selection strategy by prioritizing the geometric confidence of the predicted interface. We defined a high-confidence *“high-priority candidates”* by applying thresholds to the structure-based metrics: a Ligand Interface Predicted TM score (*ligand_iptm* ≥ 0.88) to ensure high-quality contact predictions, and a Complex predicted LDDT score (*complex_pLDDT* ≥ 0.85) to ensure overall structural integrity. This robust filtration reduced the library to 18 top-ranking candidates (Figure S2). The *ligand_iptm* and *complex_pLDDT* values for all 18 “*high-priority candidates*” are reported in Table S2. These 18 hits, along with the positive control SU056 (retained despite its relatively lower confidence scores), were advanced to 10 replicates of 50 ns MD simulations. SU056 was retained as a reference despite falling below the nominal thresholds because it provides a useful internal comparator for downstream dynamic behavior and interaction patterns, thereby allowing the confidence-based filtering step to be interpreted in the context of a known YB-1 ligand.

### 2.5 Assessment of Binding Stability

To assess the dynamic stability of ligands in the cryptic pocket, we monitored the LigFitProt RMSD of each compound relative to its initial docked pose across the 10 independent 50 ns replicate trajectories.

LigFitProt RMSD measures the time-dependent positional deviation of a ligand within the protein binding pocket by calculating the RMSD of ligand atoms after aligning the protein backbone to a reference structure. An empirical RMSD cutoff of 6.0 Å was used to distinguish ligands that remained persistently engaged with the pocket from those showing substantial pose drift or pocket disengagement. Values above this threshold were interpreted as indicative of pronounced ligand displacement, including complete unbinding and diffusion into the solvent during the short replicate simulations.

Based on this analysis seven high-persistence ligands were identified from the initial pool of 18 candidates. These compounds maintained their conformation within the binding site, as evidenced by the median RMSD profiles calculated across ten parallel 50 ns simulations (Figure S3). In contrast, the remaining ligands exhibited large RMSD fluctuations or complete solvent exposure, consistent with transient or unstable binding behavior (Figure S3). These 7 stable candidates were subsequently advanced to 500 ns all-atom extended MD simulations and binding free-energy calculations.

### 2.6 Structural Dynamics of YB-1 Candidate Ligands

The conformational stability of SU056 and the seven lead candidates within the YB-1 cryptic binding pocket was evaluated by monitoring their LigFitProt RMSD profiles over 500 ns of production MD simulations. In addition to the reference inhibitor SU056, ligands F0015–0358, F1822–0108, and F3382–0454 demonstrated high persistence, among the other four hits, maintaining their integrity within the binding site throughout the trajectory (Figure S4). Interestingly, F3382–0454 underwent a significant conformational shift by “flipping” its position within the first ∼100 ns, before reaching a stable equilibrium state that was maintained for the remainder of the simulation.

To characterize representative binding modes, trajectory frames were clustered using RMSD-based clustering. The dominant clusters for SU056, F0015–0358, F1822–0108, and F3382–0454 accounted for 72.9%, 55.4%, 76.7%, and 71.6% of the total of 10,000 frames across the 500 ns MD, respectively, indicating high convergence of these ligands within the binding groove (Table 1). It is important to note that both F0015–0358 and F1822–0108 share the benzothiazole-amide scaffold, while F3382–0454 and SU056 share a 6–6–5 tricyclic fused ring system featuring a central nitrogen heterocycle, where F3382–0454 uses this nitrogen to adhere to the binding pocket through hydrogen bonds with the backbone of the residue A120 (Figure 3). To account for ligand-size-dependent bias in Molecular Mechanics/Generalized Born Surface Area (MM/GBSA) scoring, the GBSA scores were normalized according to the number of heavy (i.e., nonhydrogen) atoms in each ligand. Notably, the ligand efficiency (LE) score of F3382–0454 was −2.07 kcal/mol per heavy atom, like that of SU056 (−2.09 kcal/mol per heavy atom; Table 1). Furthermore, the fused tricyclic architecture of F3382–0454 is shape-complementary to the aromatic groups of the binding pocket, engaging critical F74 and F85. The same behavior is observed for SU056, its 6–6–5 tricyclic architecture with F74 while further stabilizing itself through backbone hydrogen bonding with K118 (Figure 3). Although the average MM/GBSA value of F3382–0454 was less favorable than that of SU056, its combination of a stable interaction geometry, a similar LE score to SU056, sustained engagement of F74/F85, and a compact chemotype motivated its prioritization as a tractable lead scaffold.

**Table 1:**
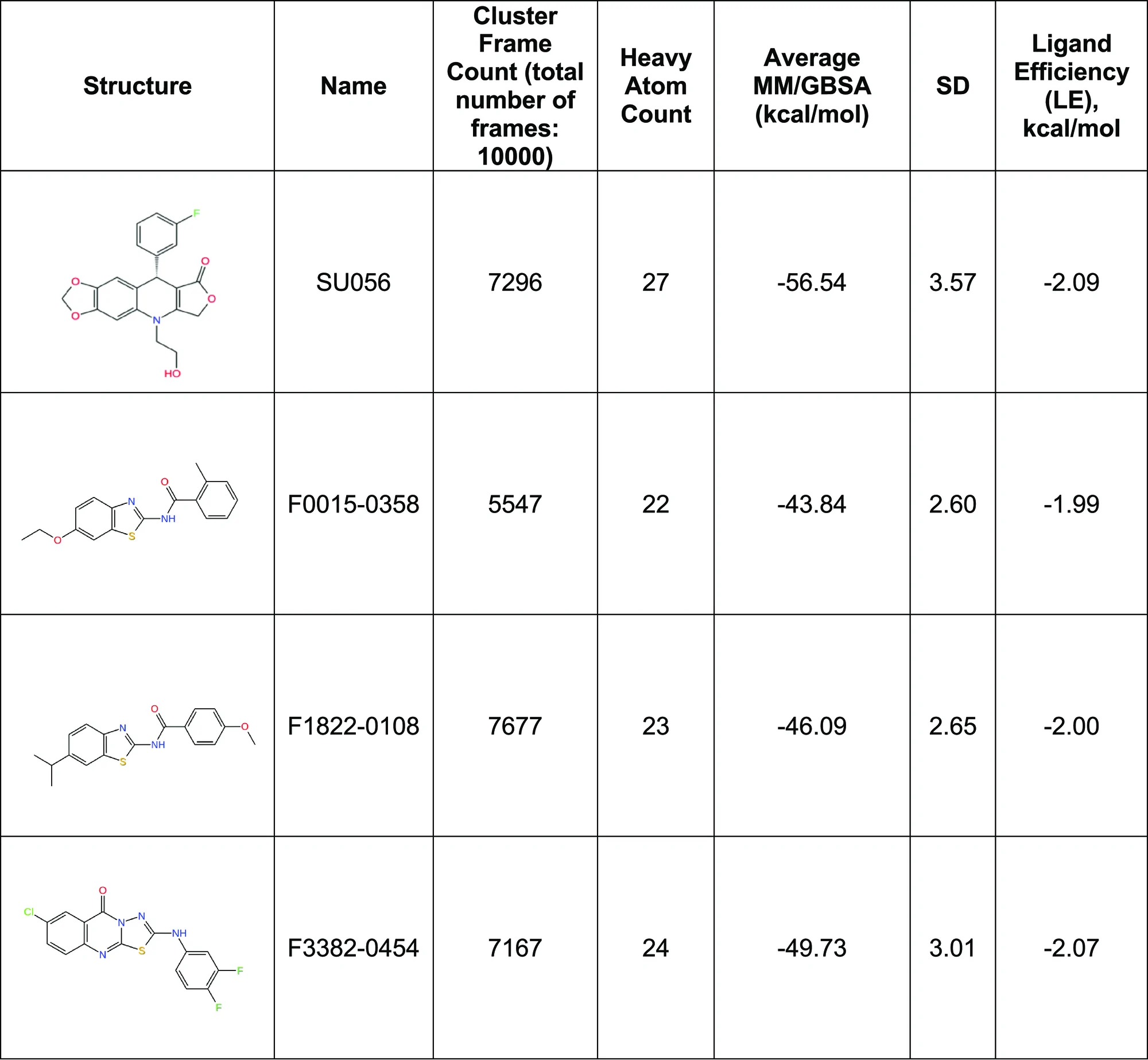
Prioritizing YB-1 Candidates^a^

a 2D structures, compound IDs, cluster frame numbers, heavy atom (non-hydrogen) count, average MM/GBSA (kcal/mol) and Ligand Efficiency (kcal/mol per heavy atom) of potential YB-1 binders.

**Figure 3:**
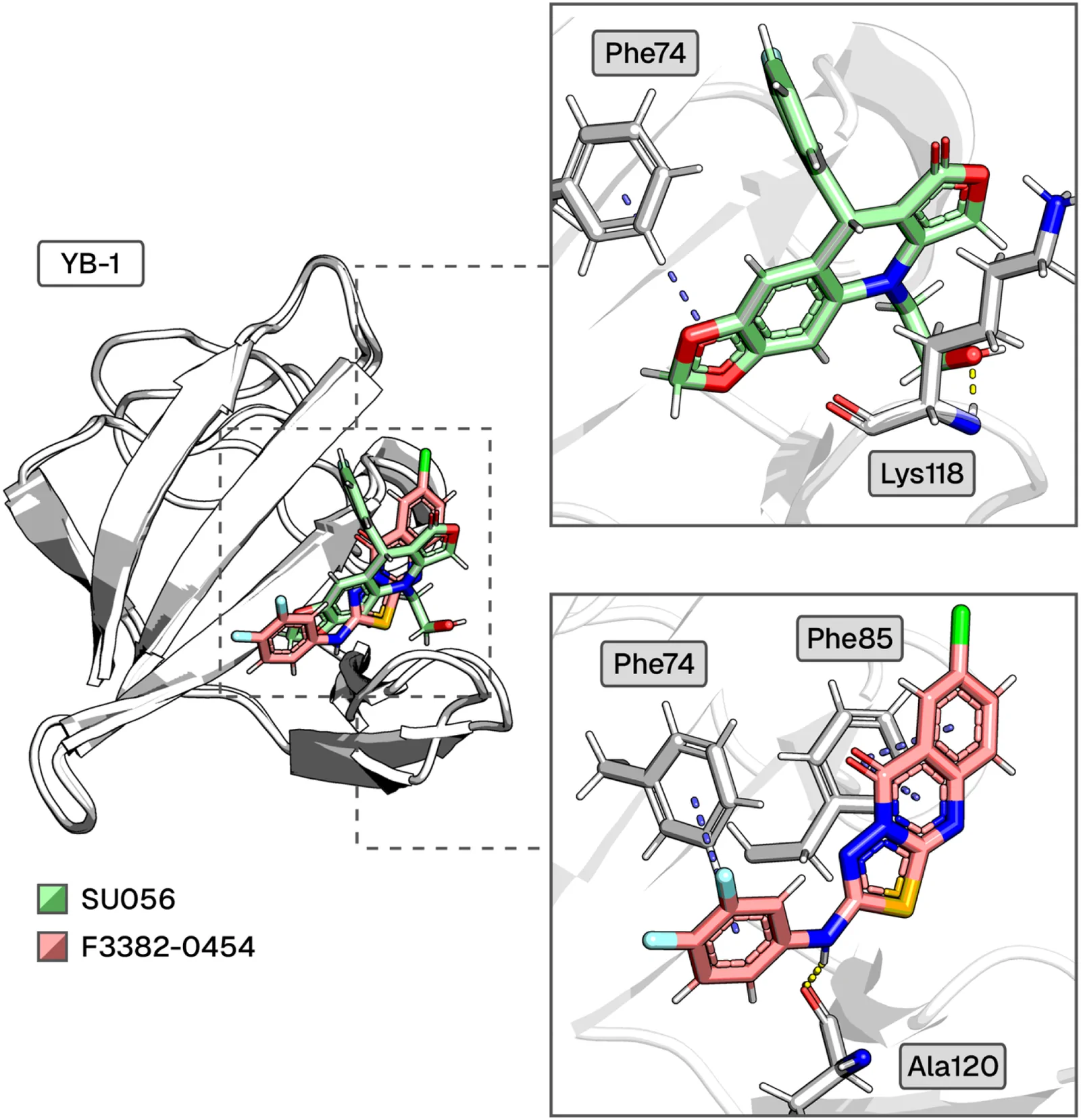
3D ligand interaction diagrams of SU056 and prioritized candidate ligand F3382–0454 with YB-1. 3D profile of SU056 in the YB-1 cryptic pocket is given in pale green on the top-right and 3D interaction profile of F3382–0454 is given in salmon on the lower right.

The interaction timeline of SU056 during the 500 ns MD simulations revealed a relatively stable binding profile, primarily maintained by residues K118, F85, and F74, all of which maintained consistent interaction occupancy throughout the collected trajectories (Figure 4A, 4B; top-left). K118 emerged as the most significant residue, contributing to interactions with SU056 through both backbone carbonyl hydrogen bonds and side chain π-cation interactions (Figure 4A; top-left). Nonetheless, both base-stacking phenylalanine residues, F74 and F85, consistently maintained π-π stacking interactions with SU056 (Figure 4A; top-left). While the core of SU056 remained fixed around aromatic residues, peripheral residues, such as E121 and E117, showed high water-bridge occupancy.

**Figure 4:**
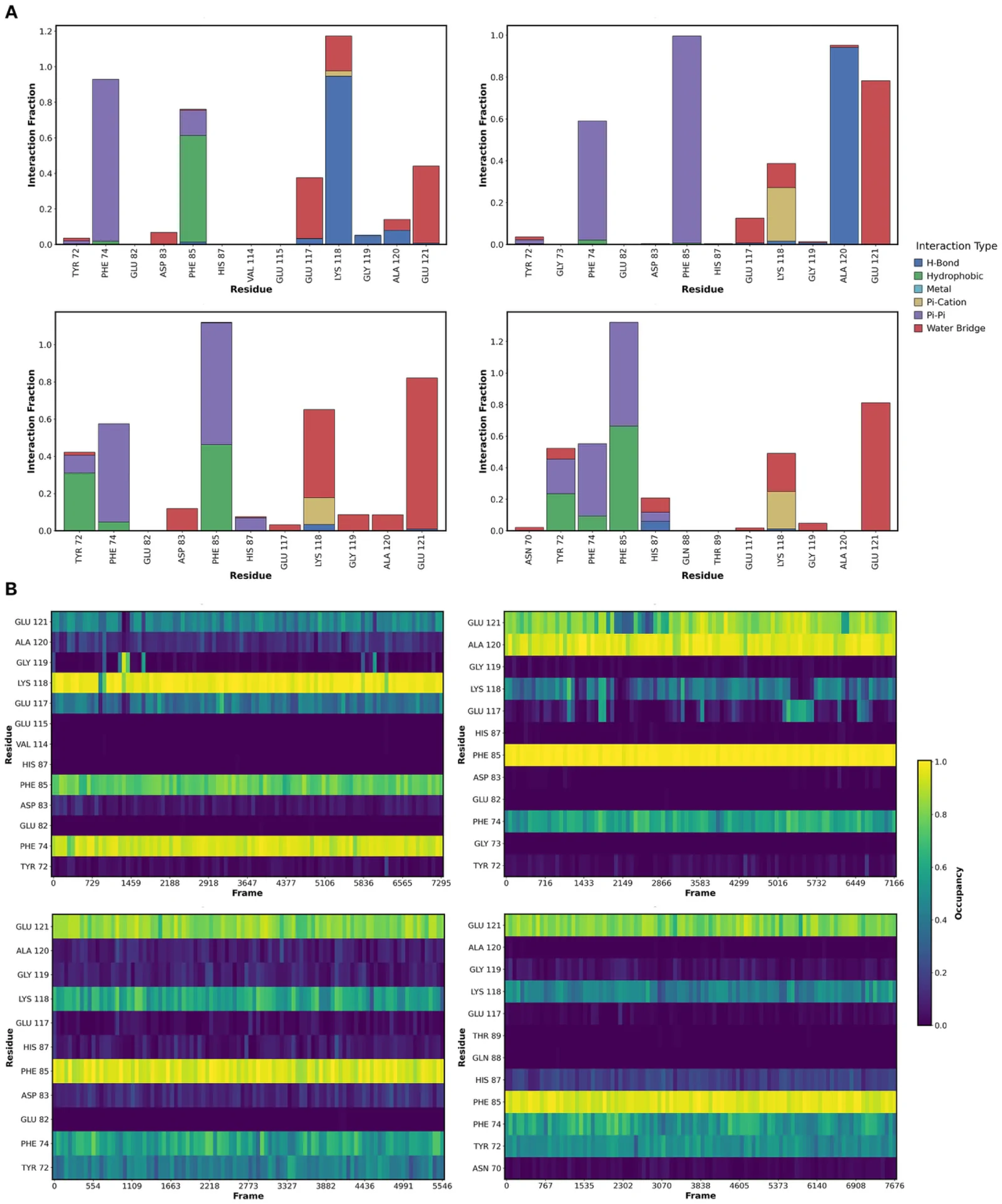
Time-dependent protein–ligand interaction summary of the clustered MD simulations. To characterize representative binding modes, trajectory frames were clustered using RMSD-based clustering. The dominant clusters for SU056, F3382–0454, F0015–0358 and F1822–0108 accounted for 72.9%, 71.6%, 55.4% and 76.7% of the total 10,000 frames throughout the 500 ns MD simulations, respectively. (A) Per-residue interaction fraction for each ligand, stacked by interaction type. Panels: SU056 (top-left), F3382–0454 (top-right), F0015–0358 (bottom-left), F1822–0108 (bottom-right). (B) Time-dependent protein–ligand contact heatmap for the same four ligands in the same panel layout.

The prioritized candidate, F3382–0454, effectively occupies the binding groove after approximately 100 ns of relaxation. F3382–0454 was prioritized as the most promising scaffold for follow-up, based on its persistent pocket occupancy, convergence of the dominant MD cluster, and ligand-efficiency value comparable to that of SU056. It is actively anchored by a hydrogen bond to the backbone carbonyl of A120 residue and by π-π stacking interactions with F74 and F85, just as in SU056 (Figure 3; Figure 4A; top-right). This interaction is maintained throughout the MD simulations after the ligand has stabilized itself (Figure 4B; top-right).

F0015–0358, one of the two benzothiazole-amide ligands, occupies the same cryptic groove as SU056 and F3382–0454 but contacts a different set of residues (Figure 4A, 4B; bottom-left). F85 contacts the ligand in more than 90% of frames through mixed hydrophobic and π-π stacking interactions, and F74 forms π-π stacking interactions with it in roughly 55% of frames, as already observed for SU056. Unlike SU056, F0015–0358 does not form hydrogen-bond directly with K118; K118 is contacted through water bridges in roughly half of the frames (Figure 4A; bottom-left). Y72 forms π-π stacking interactions with the ligand in about 45% of frames, an interaction not seen for SU056 or F3382–0454. E121 makes a water bridge interaction to the ligand in more than 80% of frames, the same C-terminal water-mediated contact found for all four compounds (Figure 4B; bottom-left).

F1822–0108, the second benzothiazole-amide hit, makes contacts similar to F0015–0358 but also reaches residues in the vicinity of the pocket (Figure 4A, 4B; bottom-right). F85 contacts the ligand in more than 90% of frames (mixed hydrophobic and π-π stacking), F74 in roughly 55%, and Y72 in about 50%, so both benzothiazole-amides recruit the same aromatic anchors F85–F74–Y72. K118 is again contacted through water bridges rather than a direct hydrogen bonding, and E121 forms a water bridge in about 80% of frames (Figure 4A; bottom-right). F1822–0108 also makes low-occupancy contacts with N70, Q88 and T89 on the vicinity of the pocket (Figure 4A, 4B; bottom right); the other three ligands do not contact with these residues.

### 2.7 Assessment of Energetic Hot Spots in the Cryptic Pocket from Per-Residue MM/GBSA Decomposition and Alanine-Scanning

To determine which cryptic-pocket residues contribute most strongly to ligand binding, an *in silico* alanine-scanning study was performed on six pocket residues: W65, F74, F85, H87, K118 and E121. The choice of these six residues is supported by three independent lines of prior evidence and by the wild-type (WT) contact analysis from the present MD trajectories (Figure 4). El Hage et al.^14^ defined the YB-1 CSD cryptic-pocket lining as W65, Y72, F74, F85, H87, K118 and E121 from a combined NMR chemical-shift perturbation and MD-pocket-detection analysis and identified K118 and F85 as the gating residues whose cation-π contact controls opening of the pocket. Yang et al.^9^ subsequently solved the structure of the YB-1 CSD bound to single-stranded RNA and, using fluorescence-polarization mutagenesis, showed that alanine substitutions at W65, F74, F85, H87, and K118 abolish detectable RNA binding. These findings established W65, F74, F85, and H87 as key aromatic π-stacking residues on the CSD recognition surface, while K118 functions as an electrostatic anchor for the RNA backbone. Zou et al.^18^ independently confirmed the involvement of the same aromatic residues in crystal structures of the CSD bound to 8-mer RNA and further demonstrated, using isothermal titration calorimeter (ITC), that W65A, F74A, F85A, and H87A destabilize RNA binding.^18^ Although Y72 and D83 have been implicated in the broader pocket environment, they were not included in the alanine scanning because they are not part of the experimentally validated mutation set reported by Yang et al.^9^ and Zou et al.^18^ and because their direct ligand contacts in the present WT trajectories were intermittent rather than persistent (Figure 4A). By contrast, E121 was retained because it is located at the C-terminal end of the cryptic pocket, as defined by El Hage et al.,^14^ and formed persistent water-mediated contacts with all ligands in the WT simulations (Figure 4A).

Thus, the selected six residues collectively represent the principal functional elements of the pocket: the gating pair K118/F85, the aromatic recognition surface formed by W65, F74, F85, and H87, and the C-terminal pocket cap represented by E121. Alanine-scanning was performed independently for each of the four protein–ligand complexes, namely SU056, F3382–0454, F0015–0358, and F1822–0108, resulting in a total of 24 mutant systems. Each of these 24 mutant complexes was generated individually rather than substituting the side chain on the WT model, every mutated residue in CSD sequence was cofolded *de novo* with its ligand by Boltz-1, the top-ranked complex was resimulated for 500 ns all-atom MD simulation and MM/GBSA was calculated under the same WT protocol. Cofolding each mutant separately lets the pocket relax around the substitution, so the resulting ΔΔ*G* captures induced-fit reorganization rather than the loss of a single contact from a fixed WT pose.

To attribute the WT MM/GBSA binding free energy to an individual CSD residue, per-residue energy decomposition was computed using the dominant cluster from each 500 ns WT trajectory, in which each protein residue was rescored in complex with the ligand (Figure 5A). Among the evaluated residues, four residues (W65, Y72, F74 and F85) consistently showed strong favorable contributions to ligand binding, with each residue contributing more favorably than −10 kcal/mol across all four ligands. F85 is the largest single contributor in the three complexes, namely F3382–0454 (−19.1 kcal/mol), F1822–0108 (−16.4 kcal/mol), and F0015–0358 (−16.3 kcal/mol), and the second-largest contributor in SU056 complex (−20.7 kcal/mol), where W65 showed the strongest contribution (−22.7 kcal/mol), Figure 5A.

**Figure 5:**
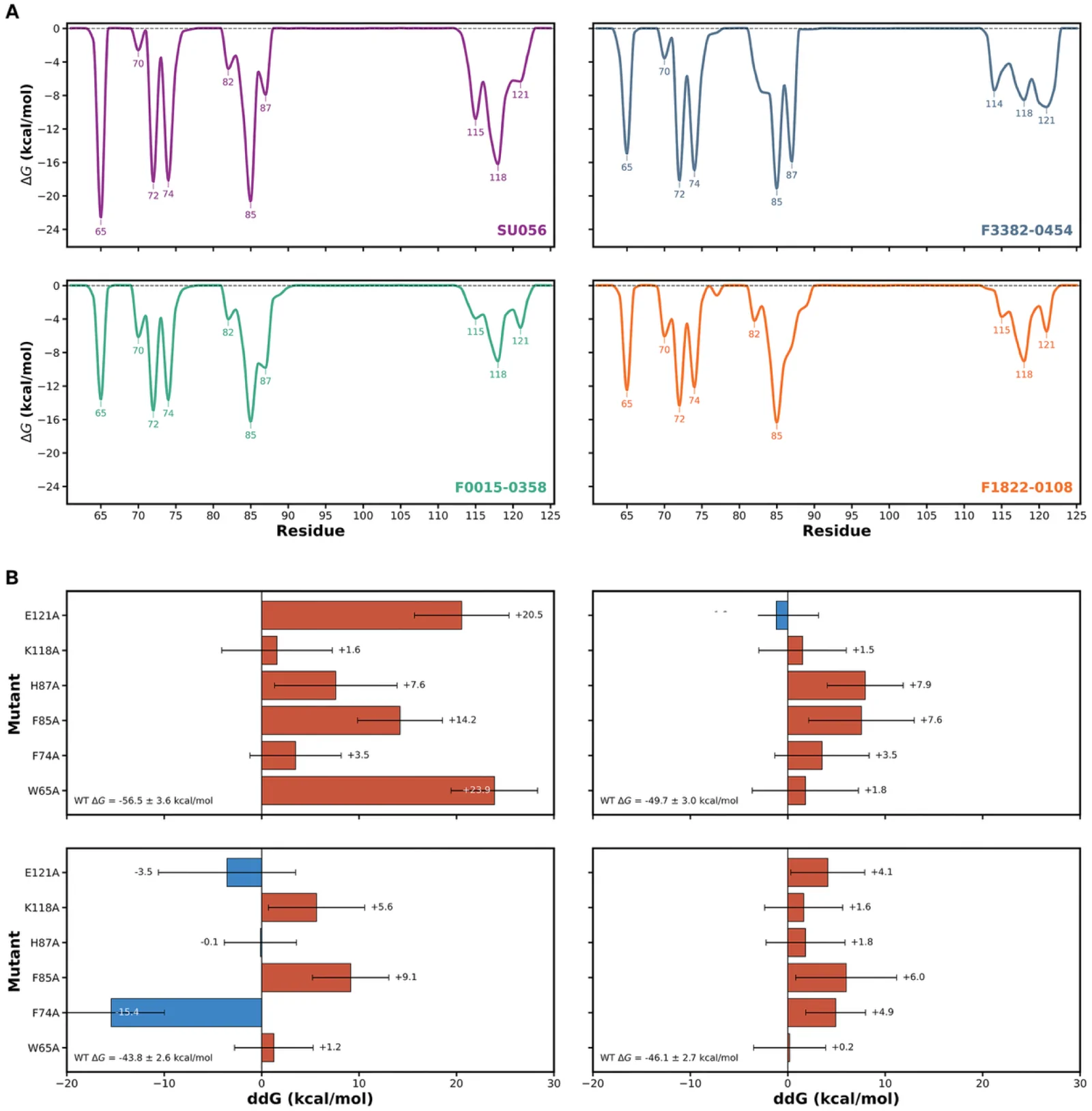
Energetic decomposition and alanine-scanning of the YB-1 CSD cryptic pocket for hit compounds. (A) Per-residue MM/GBSA decomposition over the dominant cluster from each of the 500 ns WT trajectories for the 4 ligands reported. (B) MM/GBSA alanine-scanning and ΔΔ*G* calculations for the same four ligands at six pocket residues (W65, F74, F85, H87, K118, E121). ΔΔ*G* = Δ*G*_*mutant*_ – Δ*G*_*WT*_; positive (red) values destabilize the complex, negative (blue) values stabilize it. The mean WT MM/GBSA binding free energy and ± SD are shown below each panel. The *x*-axis range is shared across the four subpanels for direct comparison. Panel positions in (A) and (B) follow SU056 top-left, F3382–0454 top-right, F0015–0358 bottom-left, F1822–0108 bottom-right.

Y72 showed consistently favorable contributions across the four ligand complexes, ranging from −14.4 to −18.4 kcal/mol, while F74 contributed between −12.1 and −18.2 kcal/mol. Together with F85, these two aromatic residues therefore represent the major contributors to the per-residue binding energy profile in all four complexes (Figure 5A). W65 also made favorable contributions, ranging from −12.5 to −22.7 kcal/mol. However, its contribution was more variable in the three prioritized ligand complexes, with standard deviations of approximately 8–10 kcal/mol, compared with a much smaller standard deviation of 2.5 kcal/mol in the SU056 complex (Figure 5A). This suggests that SU056 maintains a relatively more stable and persistent π–π stacking interaction with W65, whereas the three candidate hit compounds interact with W65 more intermittently during the simulations (Figure 5A). K118 contributed −16.2 kcal/mol to SU056 binding, which was nearly twice as favorable as its contribution to the three hit compounds, where the values ranged from −8.5 to −9.0 kcal/mol (Figure 5A). This stronger contribution is consistent with the direct backbone hydrogen bond and side chain cation−π interaction formed between K118 and SU056. In contrast, the three prioritized compounds appear to engage K118 mainly through water-mediated contacts (Figures 4A and 5A). Notably, H87 showed a particularly strong contribution to F3382–0454 binding, with a value of −15.9 kcal/mol, compared with −7.2 to −9.8 kcal/mol for the other three ligands. This enhanced contribution likely reflects the extended interaction network formed by F3382–0454 within the F85/H87/A120 subpocket (Figure 3). It should be noted that per-residue decomposition provides residue–ligand pairwise interaction energies calculated in implicit solvent. Therefore, these values should be interpreted as local energetic contributions rather than as a strict mathematical partitioning of the total holo MM/GBSA binding free energy. Consequently, the individual per-residue contributions are not expected to sum exactly to the total WT binding free energy.

The alanine-scanning analysis revealed distinct residue-dependence patterns among the four ligand complexes (Figure 5B). For SU056, three mutations produced pronounced destabilizing effects: W65A increased the calculated binding free energy by +23.9 kcal/mol, E121A by +20.5 kcal/mol, and F85A by +14.2 kcal/mol. These large positive ΔΔG values indicate that W65, E121, and F85 are critical contributors to SU056 binding. In contrast, F3382–0454 showed a more moderate sensitivity to alanine substitution. The strongest destabilizing mutations were F85A (+7.6 kcal/mol) and H87A (+7.9 kcal/mol), suggesting that this ligand depends mainly on contacts within the F85/H87 region. By comparison, K118A had only a minor effect on F3382–0454 binding (+1.5 kcal/mol), while E121A was slightly stabilizing (−1.2 kcal/mol), indicating that these residues are not essential stabilizing determinants for this complex. The two benzothiazole-amide prioritizing compounds displayed the flattest alanine-scanning profiles. For F0015–0358, only F85A produced a destabilization greater than +9 kcal/mol, with a ΔΔG value of +9.1 kcal/mol. For F1822–0108, the largest destabilizing effect was also observed for F85A, but the magnitude was relatively modest (+6.0 kcal/mol) (Figure 5B). All remaining mutations caused changes within ± 6 kcal/mol relative to the WT complex for both compounds, suggesting that their binding is distributed over multiple weak-to-moderate contacts rather than being dominated by a single residue. Among all tested residues, F85 was the only position whose mutation destabilized every ligand complex, with ΔΔG values ranging from +6 to +14 kcal/mol across the four ligands (Figure 5B). This finding is consistent with the contact analysis, where F85 remained in contact with each ligand in more than 90% of the clustered trajectory frames (Figure 4), as well as with the per-residue energy decomposition, where F85 ranked as either the largest or second-largest energetic contributor in every system (Figure 5A). Together, these results identify F85 as a conserved binding hotspot within the CSD pocket. By contrast, K118A showed ligand-dependent effects. This mutation destabilized F0015–0358 by +5.6 kcal/mol but had only minimal effects on the other three complexes, which remained within +1.5 to +1.6 kcal/mol of the WT binding free energy (Figure 5B). This difference can be explained by the interaction patterns observed in the structural analysis. SU056 forms direct hydrogen-bonding interactions with K118, whereas F0015–0358 and F1822–0108 interact with K118 mainly through water-mediated contacts. Because these water-bridged interactions are less dependent on the intact lysine side chain, they may be partially preserved during the MD simulations even after replacement of lysine with alanine (Figure 3).

Two of the six ΔΔG values calculated for F0015–0358 were negative rather than positive: F74A showed an apparent stabilization of −15.4 kcal/mol, and E121A showed a smaller apparent stabilization of −3.5 kcal/mol (Figure 5B). In addition, F3382–0454 showed a slightly negative ΔΔG value for E121A (−1.2 kcal/mol). However, we do not interpret these negative values as genuine improvements in binding affinity. This interpretation is based on the limitations of the end-point MM/GBSA approach used for the mutant trajectories. MM/GBSA calculations are sensitive to changes in ligand pose and protein relaxation during the simulation. As shown in Table S3, the F74A mutant trajectories had the lowest dominant-cluster occupancies, only 7.6–8.0%, for the three hit compounds F3382–0454, F0015–0358, and F1822–0108. This indicates that mutation of F74 to alanine increased conformational heterogeneity in the binding site and allowed the ligands to explore alternative binding poses. For F0015–0358, in particular, replacing F74 with alanine likely enlarges the cavity around the benzothiazole ring. During the simulation, the ligand can therefore resettle into a slightly different pose in this expanded pocket. This new pose may produce more favorable van der Waals and Generalized Born solvation terms in the MM/GBSA calculation, even though it does not necessarily represent a true increase in binding affinity. In other words, the scored mutant ensemble is not simply the WT binding mode with the F74 contact removed; rather, it reflects a different binding mode in a remodeled pocket. This is important because MM/GBSA provides an interaction-energy estimate combined with an implicit-solvent correction, rather than a rigorous thermodynamic binding free-energy. The method does not fully account for the configurational entropy associated with protein–ligand reorganization between the WT and mutant states. Therefore, if a mutation opens additional pocket volume and allows the protein–ligand complex to relax into a lower-enthalpy conformation, MM/GBSA may report an apparent stabilization, even when the true experimental ΔΔG would be small or unfavorable. The negative E121A values likely reflect a similar effect, but with a smaller magnitude. E121 is located near the C-terminal end of the pocket and interacts with F0015–0358 and F3382–0454 mainly through water-mediated contacts during the MD simulations rather than through a strong direct interaction. Removal of the E121 side chain can reorganize this water bridge and the surrounding solvent-exposed interaction network. Such local rearrangements are sufficient to shift the average MM/GBSA score by a few kcal/mol, without necessarily indicating a real improvement in ligand affinity. Overall, these results suggest that the negative ΔΔG values observed for F74A and E121A should be interpreted as MM/GBSA artifacts arising from pose drift, pocket relaxation, and solvent reorganization. In contrast, SU056 appears to bind the WT pocket relatively tightly and in a more structurally defined manner, but this also makes its binding mode more sensitive to mutation of key residues.

### 2.8 Implications for Targeting Intrinsic Disorder and Cryptic Pockets

The prioritization of F3382–0454 and the benzothiazole-amide series illustrates how a generative cofolding workflow can be applied to “undruggable” oncoproteins like YB-1. Traditional virtual screening often fails for the YB-1 CSD because it relies on static “lock-and-key” methods that do not account for its intrinsic flexibility. Our results suggest that the F85/K118 pocket is not static but dynamic and requires specific stereoelectronic features that we successfully captured here by 5D-ElectroShape combined with MolPrism to induce binding. The “equilibration flip” of F3382–0454 at initial 100 ns of the simulation shows that generative models’ protein folding algorithms can suggest realistic starting poses, but full binding is achieved only when protein flexibility is considered in MD simulations. This hierarchical workflow, starting from library design to generative modeling and MD, offers a scalable and reproducible approach for targeting other transient, cryptic protein interfaces in cancer research.

### 2.9 Paralog Selectivity Considerations

The CSD is shared by the human Y-box family (YB-1, YB-2, YB-3) and by LIN28A and LIN28B, and cross-paralog activity is a reasonable concern for any ligand engaging the CSD. MAFFT alignment of the canonical CSDs (Figure S5) shows that the YB-1 and YB-3 CSDs are 100% identical, that YB-2 differs by three conservative substitutions outside the cryptic pocket (95.4% sequence identity), and that the fingerprint residues described by El Hage et al.^14^ (W65, F74, F85, H87, K118, E121) are conserved across the full Y-box family and across LIN28A and LIN28B. The LIN28A and LIN28B cation-π gates appear at F73/K102 and F63/K92, respectively. Within the Y-box family, sequence-level discrimination of the cryptic pocket is not achievable. Engaging both YB-1 and YB-3 has therapeutic rationale, where YB-3 substitutes for YB-1 in global mRNA binding in YB-1-depleted cells,^19^ and the reference inhibitor SU056 depletes both YB-1 and YB-3 in glioblastoma models and potentiates immune-checkpoint blockade through the CHK2–YB-1/YB-3 hub.^20^ Reproductive toxicity is the main concern, since compound heterozygosity for *Ybx2* and *Ybx3* in mice causes male sterility through defective postmeiotic spermatid differentiation.^21^

Discrimination between the Y-box and LIN28 CSDs is also not residue-driven but stems from the loops that shape the surface around the conserved fingerprint residues. Relative to the Y-box CSD, the LIN28 CSD has a seven-residue insertion in the β2−β3 loop at the entrance to the cryptic cavity and a four-residue deletion in the β4−β5 loop, with β-strand boundaries taken from the YB-1 secondary structure of PDB 6LMS^10^ overlaid on the alignment (Figure S6). SiteMap analysis of the LIN28 CSD assigns its two best subpockets scores of 0.63 and 0.58, against 0.90 for the LIN28 zinc-knuckle cavity, which has no Y-box equivalent.^22^ The LIN28 CSD is ligand accessible, where ponicidin has been reported to bind LIN28B with a *K*_*D*_ = 16.87 nM by SPR, targeting the CSD let-7-binding surface.^23^ Documented LIN28 CSD ligands engage the let-7-binding surface of the domain rather than a deep cryptic β-barrel cavity equivalent to the YB-1 site^22,24,25^ and whether the chemotypes prioritized here cross-react with the LIN28 CSD let-7 surface is an empirical question that biophysical validation campaigns can resolve. The deep cryptic β-barrel cavity that defines the YB-1 target site sits within an OB-fold β-barrel topology and it is known that the other principal RNA-binding folds (RRM, KH, dsRBD, zinc finger) are structurally distinct from this topology,^26^ so off-target binding at the cryptic pocket to RNA-binding proteins that use these alternative folds is not expected on architectural grounds.

To test these expectations for the two closest CSD-containing paralogs, we cofolded the four prioritized ligands (SU056, F3382–0454, F0015–0358, and F1822–0108) with the LIN28A and LIN28B CSDs using Boltz-1, under the configuration used for the YB-1 runs. Of the eight paralog-ligand complexes, Boltz-1 reproduced a placement in the LIN28A/B site equivalent to the YB-1 cryptic cavity for only five, while for the remaining three it positioned the ligand outside the cavity (F0015–0358 with LIN28A, and F1822–0108 with both paralogs; Figure S6), so the benzothiazole-amide F1822–0108 did not cofold into the cryptic pocket of either LIN28 paralog. The five cryptic pocket complexes were carried into 200 ns MD simulations and their whole trajectory MM/GBSA values ranged from −24 to −37 kcal/mol (Table S4). The failure to reproduce a cryptic-pocket placement for three of the eight complexes, together with the less favorable accommodation seen in the other five, indicates that the LIN28 CSDs are poorer hosts for these chemotypes than the YB-1 cryptic pocket.

### 2.10 Methodological Limitations

The hierarchical workflow presented here combines a ligand-based descriptor stage, a generative cofolding stage, and an end-point free-energy stage. The 5D-ElectroShape descriptors are computed from a single low-energy conformer and the partial-charge term in the fourth dimension depends on the charge-assignment engine used, with atomic partial-charge discrepancies reported across conformers of the same molecule.^15,16^ Compounds outside the 70 retained cluster centroids may therefore include true binders that were not advanced into the structural pipeline.

DL protein–ligand cofolding methods as a class generate poses with strong geometric agreement but frequently fail rigorous physical validity checks on their top-ranked predictions, including steric clashes, bond-geometry violations, and stereochemistry errors,^27^ and they show an almost linear decline in accuracy as the target moves off the training distribution.^28^ The interface-confidence and per-residue confidence scores returned by these models (*ipTM*, *pLDDT*) are calibrated against the model’s structural reconstruction objective rather than against experimental binding affinity.^29^ The predicted pocket assignment can be tested directly by ^1^H-^15^N SOFAST-HMQC NMR chemical shift perturbations on the previously cataloged fingerprint residues (W65, F74, F85, H87, K118, E121).^14^

The MM/GBSA binding free energies on clustered MD frames are sensitive to the solute dielectric constant and to the GB radius set, and the configurational entropy term fluctuates strongly across MD snapshots and is therefore conventionally omitted in the standard implementation.^30,31^ The reported MM/GBSA scores rank scaffolds against the SU056 reference under the same protocol but are not interpreted as absolute binding free energies. Absolute *K*_*D*_ values can be obtained from SPR on immobilized recombinant YB-1 ^11,13^ or from ITC, which has been applied to a YB-1 cryptic-pocket inhibitor.^14^

The alanine-scanning MM/GBSA inherits the same end-point-method approximations and additionally neglects the configurational-entropy change between the WT and mutant ensembles, which is conventionally omitted because of the slow convergence of the normal-mode treatment in MM/PBSA and MM/GBSA.^32^ Pose drift on the mutant trajectory can produce apparent “gain-of-affinity” outputs (negative ΔΔ*G*) that do not reflect a real increase in binding. The per-residue MM/GBSA is computed here as a residue–ligand pairwise interaction energy under the implicit-solvent model and does not represent a closed-form decomposition of the WT MM/GBSA score. Both readouts can be tested experimentally by expressing recombinant alanine mutants of the six scanned positions and measuring ΔΔ*G* by MST or SPR against the WT and ^1^H-^15^N HMQC CSP mapping on the same residue set provides residue-resolved direct evidence of ligand contact that does not depend on the MM/GBSA partitioning.

### 2.11 Path to Experimental Validation

The screening framework reported in this study operates on computational models, and biophysical or cellular confirmation of the predicted binding modes is not presented in the current work. Additional orthogonal experimental validation to the three prioritized candidates (F0015–0358, F1822–0108, and F3382–0454), using SU056 as an internal benchmark is required. The biophysical assays that would confirm direct binding (MST, CETSA, SPR and NMR) are identified alongside the corresponding limitations in the preceding subsection; the functional cellular validation that would follow confirmed binding is outlined here. Functional cellular consequences can be tracked using sequential reporter assays, such as the *E2F1*-luciferase, γ-globin AlphaScreen, and TruHits systems^33^ alongside MT-bench high-content imaging screen, which visualizes endogenous mRNA enrichment on microtubule-tethered YB-1.^14^ Downstream transcript and protein readouts would monitor targets like *EGR1*, *MDR1*, *CD44*, and *VEGFA*.^13,33,34^ The binding of our proposed candidates to the cryptic-pocket itself can be tested by site-directed mutagenesis to the pocket-lining residue and single substitutions in recombinant YB-1 CSD assayed by MST or SPR should show a loss-of-binding signature, and the same mutants in the cellular reporter should show functional rescue or abrogation.

## 3 Conclusions

In this study, we propose a hierarchical workflow that combines chemical-space compression by 5D-ElectroShape with Boltz-1 generative cofolding and MD refinement to prioritize candidate ligands for the “undruggable” YB-1 CSD, including chemotypes that classical docking may have missed. Our results indicate that, for transient interfaces such as the YB-1 CSD cryptic pocket, dynamic structural templates are needed for accurate molecular recognition. Among the prioritized candidates, F3382–0454 reproduces the binding interactions of the known inhibitor SU056 and reaches a comparable computed binding free-energy, which makes it a tractable scaffold for further medicinal chemistry optimization. The workflow is computational throughout, and its 5D-ElectroShape, Boltz-1 and MM/GBSA stages each carry the assumptions set out in methodological limitations; the prioritized compounds are therefore hypotheses that require experimental validation, by *in vitro* binding measurements and cellular activity assays, before they can be described as inhibitors. With that validation in place, the workflow is not exclusive to YB-1 and could be applied to other transient, cryptic interfaces across the human oncoproteome.

## 4 Methods

### 4.1 Library Preparation and Descriptor Generation

A focused library of 11,594 molecules was curated from the following libraries: ChemDiv RNA-Protein Interaction Inhibitors Library with 2225 molecules (https://www.chemdiv.com/catalog/focused-and-targeted-libraries/rna-protein-interaction-inhibitors-library), LifeChemicals RNA-associated Protein Screening Library with 6569 molecules (https://lifechemicals.com/screening-libraries/targeted-and-focused-screening-libraries/rna-associated-protein-screening-library) and the Enamine RNA G-quadruplex Library with 2800 molecules (https://enamine.net/compound-libraries/targeted-libraries/rna). We generated molecular descriptors using our custom open-source Python implementation of the 5D-ElectroShape algorithm (https://github.com/lalehanoktay/electroshape), which adheres to the protocol validated by Armstrong et al.^15,16^ Our implementation of the 5D-ElectroShape formalism is a descriptor generation method that closely follows the Armstrong et al. method, which is based on the statistical moments of atomic distributions. Our method constructs an 18-digit vector by calculating the mean, variance, and skewness of the distances of atoms in 5D space, extensively reported in Armstrong et al. 2011.^16^

### 4.2 Clustering and Selection of Focused Screening Library

The 5D-ElectroShape features were processed using the MolPrism toolkit (Molecular Clustering and Analysis Toolkit) (https://github.com/DurdagiLab/MolPrism-Molecular-Clustering-and-Analysis-Toolkit). We used an automated k-means clustering module to determine the optimal partitioning of the data set, evaluating both the Elbow method and Silhouette scores. This partitioning resulted in k = 7 distinct clusters based on 5D-ElectroShape properties. The top 10 representative centroids were extracted from each cluster, resulting in a set of 70 focused compounds.

The purpose of this compression step was to preserve broad descriptor-space diversity in a computationally tractable subset for downstream structure-based evaluation. In this context, the selected representatives should be interpreted as diversity centroids of the screened library.

### 4.3 Conventional Docking Benchmark

The YB-1 CSD receptor was prepared from PDB 6KUG^18^ using the Protein Preparation Wizard of the Schrödinger Molecular Modeling Suite, with the cocrystallized 4-mer RNA excised. Four docking grids were generated for Glide/SP^17^ with centroids placed on the four RNA-contact subsites described by Yang et al.^9^ F85/K118 (A2 subsite), W65/F74/D83 (U3 subsite), W65/N67 (C4 subsite) and H87/E121 (C1 subsite). SU056 was docked into each grid under the Glide/SP scoring function, and the top-ranked pose from each grid was used as the starting structure for three independent 1 μs Desmond MD replicas (12 trajectories in total), run under the protocol reported previously.^35^ For each frame, the center of mass (CoM) distance between SU056 and the F74 side chain CoM, and between SU056 and the K118 side chain CoM, was recorded.

### 4.4 Deep Learning (DL)-Based Complex Prediction

Protein–ligand complex structures were predicted using Boltz-1 (MIT) using the Boltz predict module.^36^ For each of the 71 ligands (70 cluster representatives + SU056), a YAML configuration was generated pairing the ligand SMILES with the YB-1 sequence. Predictions utilized the MMseqs2MSA server.

The YB-1 CSD (UniProt P67809, residues 61–125) sequence was defined as

GTVKWFNVRNGYGFINRNDTKEDVFVHQTAIKKNNPRKYLRSVGDGETVEFDVVEGEKGAEAANV

The four ligands analyzed in detail for YB-1 (SU056, F0015–0358, F1822–0108, and F3382–0454) were cofolded with the LIN28A and LIN28B CSDs. Each ligand was paired with each paralog CSD in a Boltz-1 YAML configuration and cofolded with the MMseqs2MSA server, under the parameters used for the YB-1 runs.

The LIN28A CSD (UniProt Q9H9Z2, residues 39–112) and the LIN28B CSD (UniProt Q6ZN17, residues 29–102) sequences were defined as

LIN28A: HGAGICKWFNVRMGFGFLSMTARAGVALDPPVDVFVHQSKLHMEGFRSLKEGEAVEFTFKKSAKGLESIRVTGP

LIN28B: RGTGHCKWFNVRMGFGFISMINREGSPLDIPVDVFVHQSKLFMEGFRSLKEGEPVEFTFKKSSKGLESIRVTGP

### 4.5 Statistical Analysis

To prioritize candidates for subsequent MD simulations, a structure-driven selection strategy was adopted. High-confidence candidates (designated as the “Gold Tier”) were identified using two ligand-interface-based confidence metrics. A cutoff was applied to the Ligand Interface Predicted TM score (ligand_*iptm* > 0.88) and to the Complex Predicted Local Distance Difference Test score (*complex_pLDDT* > 0.85), and complexes passing these thresholds were advanced. Typically, a *pLDDT* score exceeding 0.70 signifies a high level of confidence in the predictions, indicating that the placement of the backbone is likely accurate. When the *pLDDT* score exceeds 0.90, it indicates greater precision and closer alignment with experimental results.^29^ It must be noted that the *ligand_iptm* and *complex_pLDDT* cutoffs were used here as pragmatic ranking criteria to reduce the number of candidates entering MD, rather than as validated classification boundaries between binders and nonbinders. These thresholds were established to select complexes with confidence scores exceeding those of the known binder SU056.

### 4.6 MD Simulations

MD simulations were performed using Desmond.^37^ The systems were solvated in an orthorhombic box using the TIP3P water model^38^ and neutralized with appropriate counterions and 0.15 M NaCl salt solution, with a minimum buffer distance of 10 Å between the protein atoms and the box edges. The OPLS3e force field^39^ was used to model protein–ligand interactions. Prior to production runs, the systems were minimized and relaxed using the default Desmond equilibration protocol. Production simulations were conducted in the NPT ensemble with the temperature maintained at 310 K using the Nosé-Hoover chain thermostat^40^ and pressure at 1.01325 bar using the Martyna-Tobias-Klein barostat.^41^ The equations of motion were integrated with a time step of 2 fs using the RESPA method.^42^ Long-range electrostatic interactions were calculated using the Particle Mesh Ewald (PME) method^43^ with a cutoff radius of 9.0 Å. Frames were extracted every 50 ps. Initially, 10 independent replicate simulations of 50 ns each were run for each system by assigning random seed numbers to the initial velocities. Next, 500 ns MD simulations were performed to the filtered molecules. For the YB-1 paralogs LIN28A and LIN28B, 200 ns MD simulations were performed with a 20 ps recording interval.

### 4.7 LigFitProt and Occupancy Calculations

Ligand fit on to protein (LigFitProt) calculations were performed using an in-house TCL script. To measure ligand stability, the trajectory was first aligned to the protein Cα atoms, and the ligand’s RMSD was calculated.

### 4.8 Trajectory Clustering

Trajectory clustering was performed using the trajectory clustering script of the Schrödinger molecular modeling suite. Clusters were generated using the affinity propagation (AP) algorithm, which identifies cluster centers by passing messages between data points.^44^ In contrast to traditional clustering algorithms, AP clustering treats all data points as potential cluster centers rather than randomly selecting a small subset of cluster centers at the beginning. Messages (responsibilities and availabilities) are passed recursively until a high-quality set of cluster centers starts to appear.^44^ The similarity metric is calculated from a reference structure. Before clustering, all protein backbone atoms of the trajectory were aligned, and the clustering reference was selected as the ligand atoms. For cluster merging, a cutoff distance of 2.0 Å was selected. The resulting most crowded cluster members were saved as trajectories and submitted to subsequent MM/GBSA calculations.

### 4.9 MM/GBSA Free Energy Calculations

To estimate the binding free energy of the top candidates, the MM/GBSA method using the Prime module from the Schrödinger Molecular Modeling Suite was employed. The solvent was treated using the VSGB 2.0 continuum solvation model^45^ and the OPLS3e force field,^39^ and the ligand was constrained to a 5.0 Å radius. MM/GBSA calculations were performed on the clustered trajectories, as produced by the AP clustering, with each trajectory extracted every 5 ns.

For the YB-1 paralog LIN28A and LIN28B, MM/GBSA was performed on the whole trajectory, and frames were extracted every 2 ns, yielding 100 frames per system.

### 4.10 Per-Residue MM/GBSA Decomposition

To attribute the WT MM/GBSA binding free-energy to individual CSD residues, a per-residue decomposition was performed. The dominant trajectory cluster of each WT 500 ns MD run, as produced by the AP clustering, was used as the input ensemble. For each protein residue *i* in the CSD construct (residues 61–125), the residue and the ligand were jointly extracted with a 5 ns stride and scored with Prime MM/GBSA under the VSGB 2.0 solvation model^45^ and the OPLS3e force field,^39^ as described in the MM/GBSA free energy calculations section. This decomposition analysis is a residue–ligand pairwise interaction energy evaluated in implicit solvent rather than a closed-form per-residue partition of the holo MM/GBSA score. It captures the direct ligand interaction with each residue under the WT pose ensemble but does not include the residue’s coupling to the rest of the protein, and per-residue values are not expected to sum to the total binding free energy.

### 4.11 Alanine-Scanning Mutagenesis

Six pocket residues (W65, F74, F85, H87, K118, E121) were considered for alanine mutation. For each system, the mutant ligand–protein complex was generated *de novo* by Boltz-1 cofolding rather than by side-chain substitution on the WT model. For each of the six alanine substitutions, the YB-1 CSD sequence was edited to replace the target residue with alanine and the mutant sequence was paired with the ligand SMILES in a Boltz-1 YAML configuration. Predictions used the MMseqs2MSA server under the same parameters used for the WT runs. The top-ranked mutant model was then resimulated for 500 ns under the same protocol used for the WT runs. Average MM/GBSA value was recomputed on the dominant trajectory cluster of each mutant, the ΔΔ*G* values are calculated as

$$\Delta \Delta G=\Delta G_{mutant}-\Delta G_{WT}$$

Positive values destabilize the complex relative to the WT and negative values stabilize.

### 4.12 Multiple Sequence Alignment (MSA) and Pairwise Identity Analysis

Cold shock domain sequences were retrieved from UniProt using the canonical domain annotations: YBX1 (P67809, residues 61–125), YBX2 (Q9Y2T7, 93–163), YBX3 (P16989, 93–157), LIN28A (Q9H9Z2, 39–112) and LIN28B (Q6ZN17, 29–102). Multiple sequence alignment was performed with MAFFT v7 via the EBI MAFFT web service under default parameters^46,47^ and the percent-identity matrix returned by the service (computed by Clustal 2.1) was used directly. The alignment was rendered with ESPript 3.0 ^48^ with the secondary structure of the YB-1 CSD from PDB 6LMS^10^ overlaid above the alignment.

## Supplementary Material



## Notes

Center of mass (CoM) distance traces of SU056 to the F74 and K118 side chains during 1 μs MD replicates for the docking benchmark (Figure S1); selection strategy for the complexes generated with Boltz-1 (Figure S2); LigFitProt RMSD distributions for the 18 Gold Tier candidates across the 10 × 50 ns replicate simulations (Figure S3); LigFitProt RMSD over the 500 ns trajectories for the seven hits (Figure S4); CSD multiple sequence alignment and pairwise sequence identity matrix for the Y-box and LIN28 paralogs (Figure S5); Boltz-1 cofolded ligand placements on the LIN28A and LIN28B CSDs (Figure S6); Glide/SP docking scores and MD pocket persistence outcomes from 1 μs MD simulations for the four receptor grids (Table S1); Boltz-1 confidence scores (*ligand_iPTM* and *complex_pLDDT*) for the 18 prioritized candidates (Table S2); dominant cluster MD occupancies for the WT and alanine mutant complexes (Table S3); and MM/GBSA binding free energies against the LIN28A, and LIN28B CSDs (Table S4) (PDF)
