# Supplementary material for: Prioritizing Candidate YB‑1 Cold Shock Domain Ligands via 5D-ElectroShape and Boltz Generative Cofolding
Source: ACS Omega. 2026 Aug 14;11(33):49432–46. doi: 10.1021/acsomega.6c02840 (PMC13520429; doi:10.1021/acsomega.6c02840)
Supplement: Supplementary file 1 [file ao6c02840_si_001.pdf]

SUPPORTING INFORMATION

Prioritizing Candidate YB-1 Cold Shock Domain Ligands via 5D-ElectroShape and Boltz Generative Co-Folding

Lalehan Oktay[a,b,c], Cem Uğuz[a,b,d], Serdar Durdağı[a,b,d,e,*]

[a]Computational Biology and Molecular Simulations Laboratory, Department of Biophysics, School of Medicine, Bahçeşehir University, 34734, Istanbul, Türkiye
[b]Lab for Innovative Drugs (Lab4IND), Computational Drug Design Center (HİTMER), Bahçeşehir University, 34734, Istanbul, Türkiye
[c]Neuroscience Program, Graduate School, Bahçeşehir University, 34734, Istanbul, Türkiye
[d]Quantitative System Biology Lab, Faculty of Medicine, Biruni University, 34015, Istanbul, Türkiye
[e]Molecular Therapy Lab, Department of Pharmaceutical Chemistry, School of Pharmacy, Bahçeşehir University, 34353, Istanbul, Türkiye

SUPPORTING FIGURES

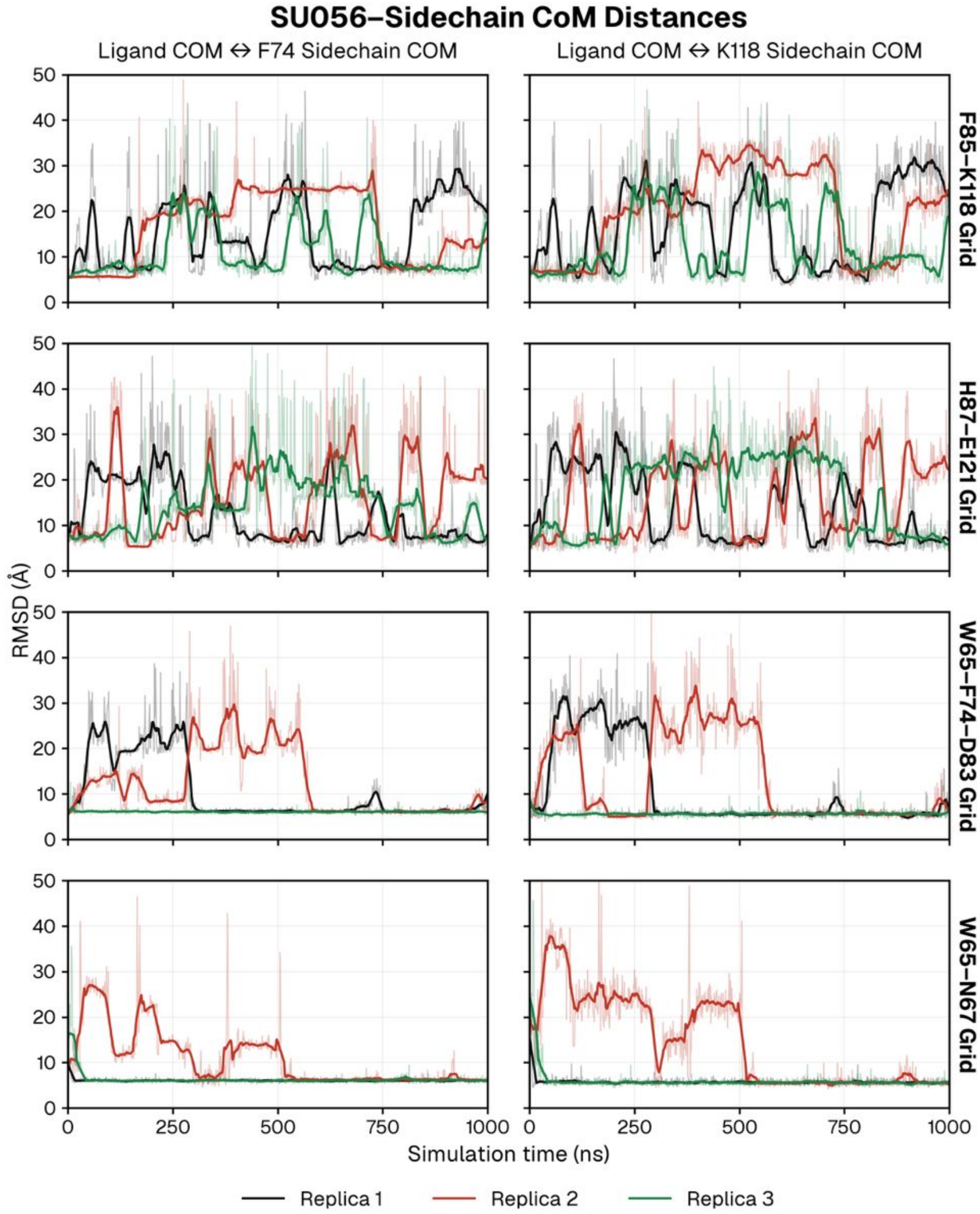

Figure S1: SU056 ligand CoM distances to F74 and K118 sidechain CoMs monitored for 3 independent 1 µs MD simulations. MD starting poses were selected from the top-ranked Glide/SP complexes on each four grids. Distance to F74 sidechain CoM (left) and to K118 sidechain CoM (right). Top to bottom: F85–K118 cryptic pocket/A2 subsite; H87–E121 / C1 subsite; W65–F74–D83 / U3 subsite; W65–N67 / C4 subsite.

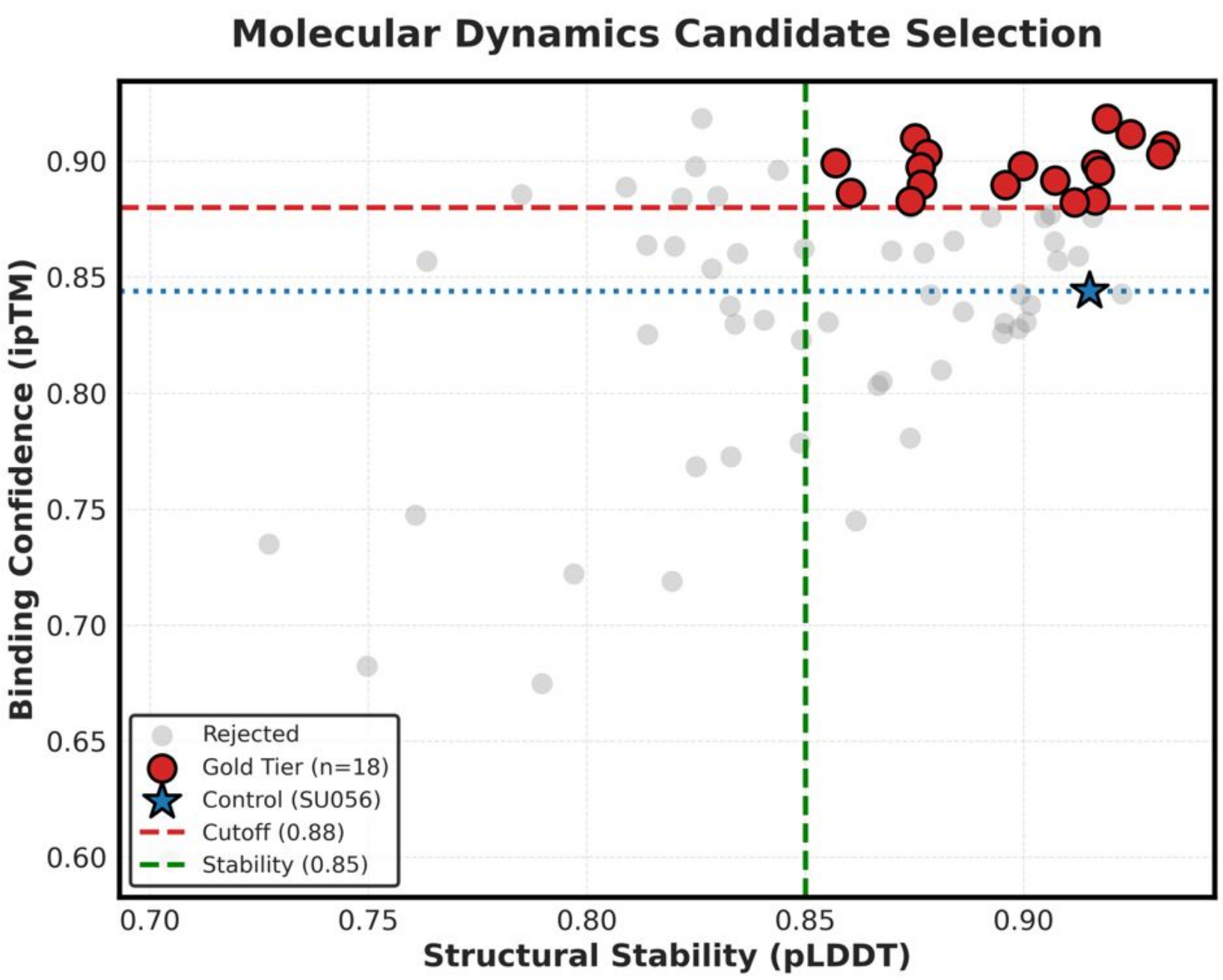

Figure S2: Selection strategy for Boltz generated complexes

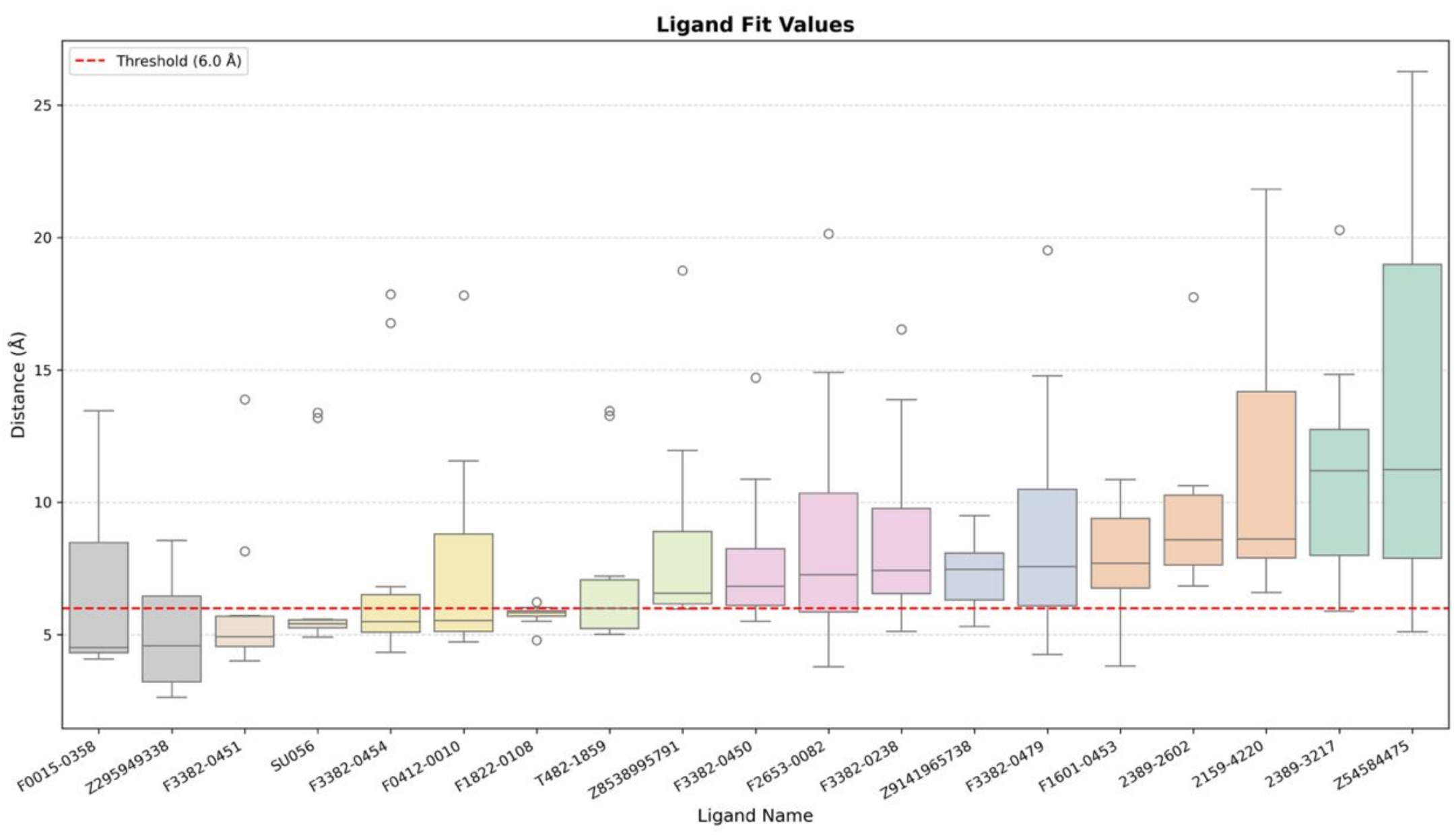

Figure S3: LigFitProt RMSD box and whisker plot for 18 prioritized 18 high-confidence candidate compounds. Calculations were extracted from 10 x 50 ns MD simulations.

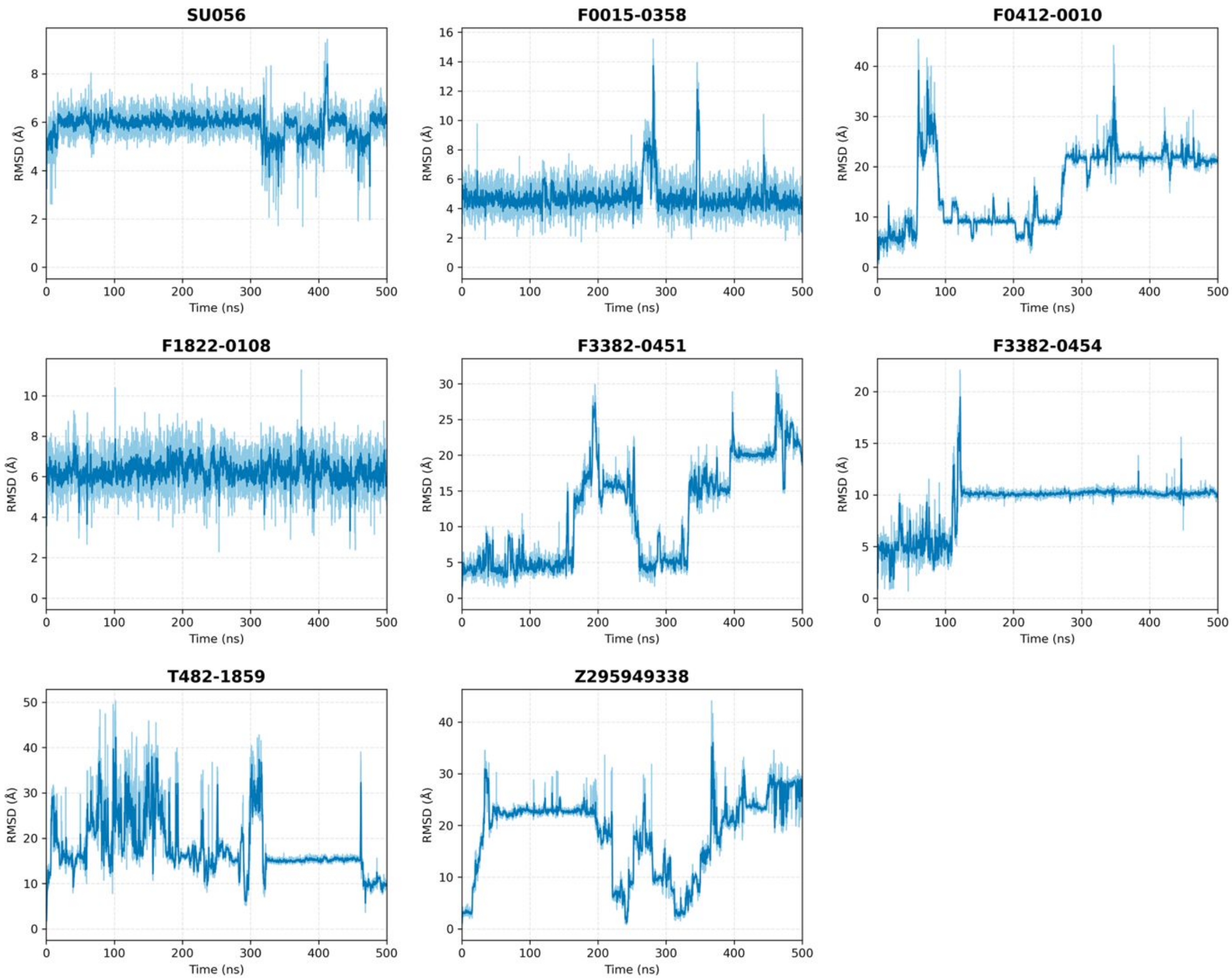

Figure S4: LigFitProt RMSD over 500 ns for top-7 ligands.

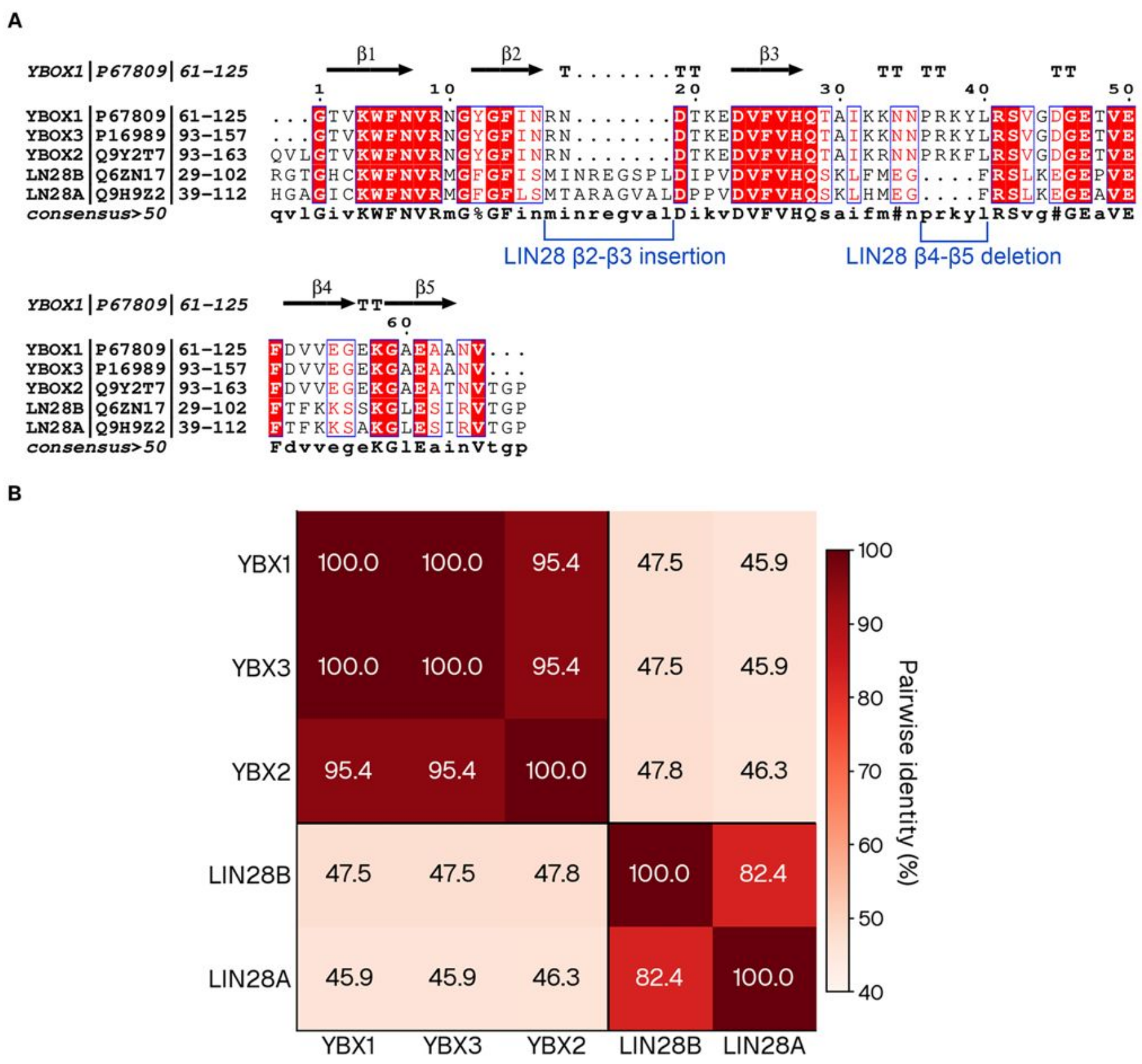

Figure S5: Cold shock domain alignment and pairwise sequence identity across the Y-box and LIN28 paralogs. (A) MAFFT v7 multiple sequence alignment of the canonical CSDs (UniProt accessions and residue ranges: YBX1 P67809, 61–125; YBX2 Q9Y2T7, 93–163; YBX3 P16989, 93–157; LIN28A Q9H9Z2, 39–112; LIN28B Q6ZN17, 29–102), rendered with ESPript 3.0 and the YB-1 secondary structure from PDB 6LMS (Zhang et al., 2020) overlaid above the alignment. Residues identical across all five sequences are shaded red; conservatively substituted positions are boxed. (B) Pairwise sequence identity (%) derived from the alignment in (A), as returned by the EBI MAFFT percent-identity matrix (Clustal 2.1).

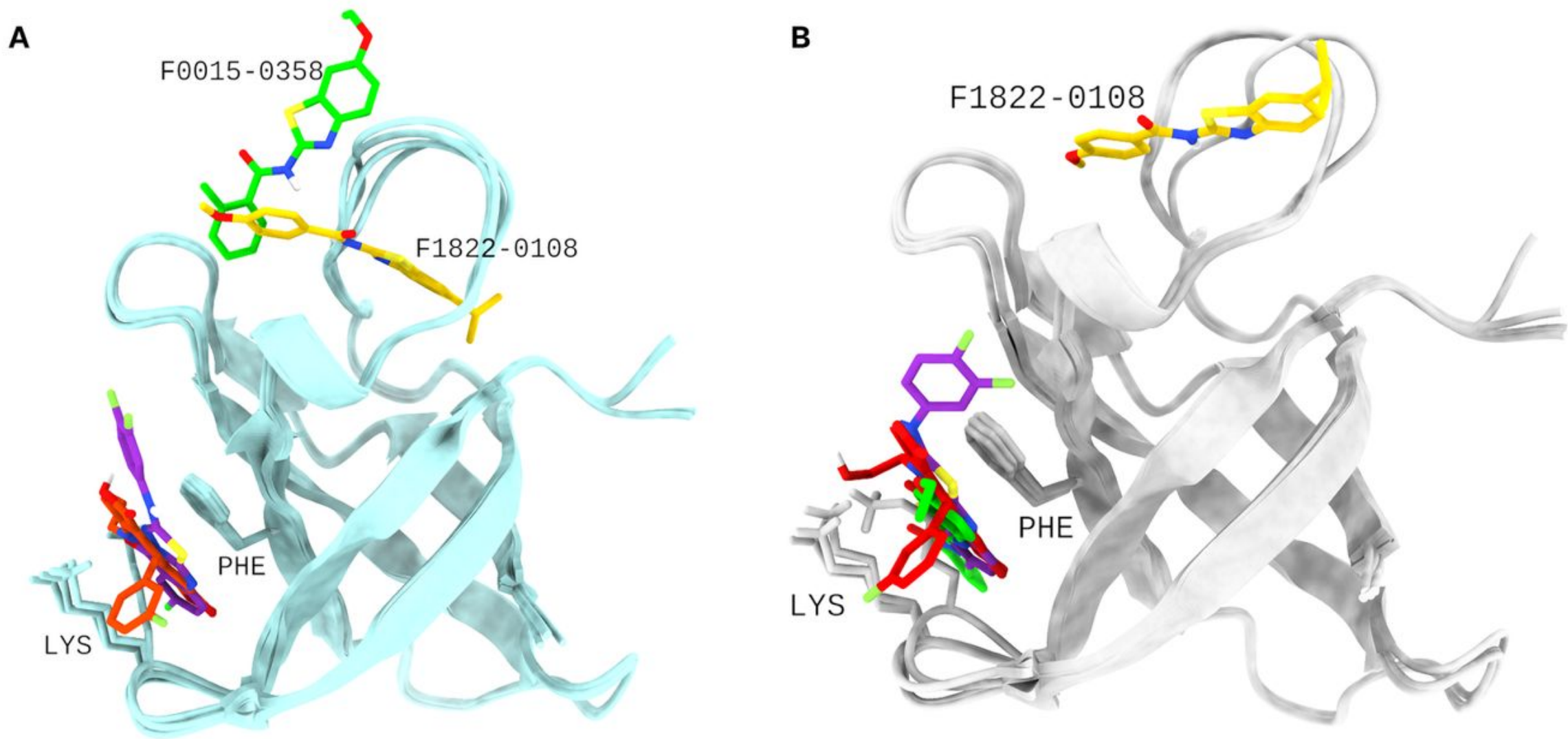

Figure S6: Boltz-1 co-folded placements of the four analyzed ligands on the LIN28A and LIN28B CSDs. (A) LIN28A CSD. (B) LIN28B CSD. For each paralog, the Boltz-1 co-folded poses of SU056, F0015-0358, F1822-0108, and F3382-0454 are shown on the LIN28 CSD.

SUPPORTING TABLES

Table S1: Glide/SP docking score versus 1 µs MD pocket-persistence outcome for each of the four receptor grids. Simulation is considered converged if SU056 maintained ~6 Å CoM distance to both F74 and K118 sidechain CoMs for the majority of the trajectory after equilibration.

Starting grid	Docking Score (kcal/mol)	Replicas with sustained contacts to F74 and K118
W65–F74–D83	−4.56	1 / 3
H87–E121	−4.43	0 / 3
F85–K118	−4.23	0 / 3
W65–N67	−3.72	2 / 3

Table S2: Boltz-1 confidence scores for the 18 Gold-Tier candidates.

Molecule ID	ligand_iptm*	complex_pLDDT*
F1822-0108	0.92	0.92
2389-2602	0.91	0.92
F2653-0082	0.91	0.88
F0412-0010	0.91	0.93
F3382-0238	0.90	0.88
2389-3217	0.90	0.93
Z54584475	0.90	0.86
F3382-0454	0.90	0.92
F3382-0451	0.90	0.90
Z295949338	0.90	0.88
F0015-0358	0.90	0.92
T482-1859	0.89	0.91
Z9141965738	0.89	0.88
F1601-0453	0.89	0.90
F3382-0479	0.89	0.86
F3382-0450	0.88	0.92
Z8538995791	0.88	0.87
2159-4220	0.88	0.91

* Both metrics are confidence estimates of the predicted interface geometry and were used here as a selection filter to reduce the number of candidates entering MD, not as predictors of binding affinity.

Table S3: Dominant trajectory-cluster occupancy for the wild-type and alanine-mutant protein-ligand complexes. Each 500 ns trajectory (10,000 frames) was clustered with the AP algorithm under the protocol used for the WT runs. The table reports the number of frames in the dominant cluster, on which the mutant MM/GBSA $\Delta\Delta G$ values in Figure 5B were computed, with the cluster occupancy in parentheses. WT dominant-cluster frame counts are reproduced from Table 1 for reference. Residue substitutions are ordered as in Figure 5.

Molecule ID	System	Dominant Cluster Frame Number	Total Frames	Occupancy Percent (%)
SU056	WT	7296	10000	73.0
	W65A	1475	10000	14.8
	F74A	1787	10000	17.9
	F85A	1058	10000	10.6
	H87A	2206	10000	22.1
	K118A	980	10000	9.8
	E121A	1679	10000	16.8
F3382-0454	WT	7167	10000	71.7
	W65A	3186	10000	31.9
	F74A	759	10000	7.6
	F85A	1817	10000	18.2
	H87A	3725	10000	37.2
	K118A	4031	10000	40.3
	E121A	7762	10000	77.6
F0015-0358	WT	5547	10000	55.5
	W65A	2154	10000	21.5
	F74A	803	10000	8.0
	F85A	903	10000	9.0
	H87A	491	10000	4.9
	K118A	4298	10000	43.0
	E121A	2453	10000	24.5
F1822-0108	WT	7677	10000	76.8
	W65A	8293	10000	82.9
	F74A	792	10000	7.9
	F85A	999	10000	10.0
	H87A	6869	10000	68.7
	K118A	9095	10000	91.0
	E121A	4854	10000	48.5

Table S4: MM/GBSA binding free energies of the analyzed ligands against the LIN28A and LIN28B CSDs. Values are the average MM/GBSA (kcal/mol) ± SD computed over the full 200 ns production trajectory, with snapshots extracted every 2 ns. N/A: Boltz-1 co-folding did not place the ligand within in the LIN28A/B site equivalent to the YB-1 cryptic cavity (Figure S6), so those complexes were not advanced to MD.

Target	Molecule ID	Average MM/GBSA (kcal)	SD
LIN28 A	SU056	-24.19	9.14
	F0015-0358	N/A	N/A
	F1822-0108	N/A	N/A
	F3382-0454	-35.09	6.70
LIN28 B	SU056	-27.61	9.76
	F0015-0358	-28.86	9.77
	F1822-0108	N/A	N/A
	F3382-0454	-37.21	7.73
